# Toxicological and pharmacological assessment of AGEN1884, a novel human IgG1 anti-CTLA-4 antibody

**DOI:** 10.1371/journal.pone.0191926

**Published:** 2018-04-04

**Authors:** Randi B. Gombos, Ana Gonzalez, Mariana Manrique, Dhan Chand, David Savitsky, Benjamin Morin, Ekaterina Breous-Nystrom, Christopher Dupont, Rebecca A. Ward, Cornelia Mundt, Benjamin Duckless, Hao Tang, Mark A. Findeis, Andrea Schuster, Jeremy D. Waight, Dennis Underwood, Christopher Clarke, Gerd Ritter, Taha Merghoub, David Schaer, Jedd D. Wolchok, Marc van Dijk, Jennifer S. Buell, Jean-Marie Cuillerot, Robert Stein, Elise E. Drouin, Nicholas S. Wilson

**Affiliations:** 1 Immuno-modulatory Drug Discovery, Agenus Incorporated, Lexington, Massachusetts, United States of America; 2 Research Biochemistry, Agenus Incorporated, Lexington, Massachusetts, United States of America; 3 Agenus Switzerland Incorporated, Basel, Switzerland; 4 Translational Biomarkers, Agenus Incorporated, Lexington, Massachusetts, United States of America; 5 Safety, Pharmacology and Toxicology, Agenus Incorporated, Lexington, Massachusetts, United States of America; 6 The Ludwig Institute for Cancer Research, New York, New York, United States of America; 7 Memorial Sloan Kettering Cancer Center, New York, New York, United States of America; 8 Agenus United Kingdom Limited, Cambridge, United Kingdom; 9 Research and Development Management, Agenus Incorporated, Lexington, Massachusetts, United States of America; 10 Research and Development Consultant, Agenus Incorporated, Lexington, Massachusetts, United States of America; Duke University School of Medicine, UNITED STATES

## Abstract

CTLA-4 and CD28 exemplify a co-inhibitory and co-stimulatory signaling axis that dynamically sculpts the interaction of antigen-specific T cells with antigen-presenting cells. Anti-CTLA-4 antibodies enhance tumor-specific immunity through a variety of mechanisms including: blockade of CD80 or CD86 binding to CTLA-4, repressing regulatory T cell function and selective elimination of intratumoral regulatory T cells via an Fcγ receptor-dependent mechanism. AGEN1884 is a novel IgG1 antibody targeting CTLA-4. It potently enhanced antigen-specific T cell responsiveness that could be potentiated in combination with other immunomodulatory antibodies. AGEN1884 was well-tolerated in non-human primates and enhanced vaccine-mediated antigen-specific immunity. AGEN1884 combined effectively with PD-1 blockade to elicit a T cell proliferative response in the periphery. Interestingly, an IgG2 variant of AGEN1884 revealed distinct functional differences that may have implications for optimal dosing regimens in patients. Taken together, the pharmacological properties of AGEN1884 support its clinical investigation as a single therapeutic and combination agent.

## Introduction

CTLA-4 is a central negative regulator of T cell function and is required to maintain immunologic homeostasis [1]. This is exemplified by CTLA-4-deficient mice and germline haploinsufficiency in humans that present with pronounced lymphoproliferative disorders, manifested by the infiltration and accumulation of T cells into lymphoid and non-lymphoid tissues [1–3]. CTLA-4 regulates activated T cell function *via* its ability to modulate co-stimulatory signals at the interface of antigen-presenting cells (APCs) and T cells, commonly referred to as the immune synapse. During T cell priming, peptide-loaded major histocompatibility complex (MHC) molecules expressed by APCs are recognized by T cells *via* the T cell receptor (TCR) [4]. In addition to TCR ligation, optimal T cell proliferation, cytokine production and effector differentiation require co-stimulatory signaling provided by CD28 [5]. CD28 is constitutively expressed on the surface of naïve CD4^+^ and CD8^+^ T cells, whereas CTLA-4 is rapidly translocated from intracellular protein stores in the trans Golgi network to the cell surface upon activation at a level proportional to strength of the TCR stimulus [6, 7]. Like CTLA-4, CD28 binds to CD80 and CD86 on the surface of activated APCs, with CD28 signaling promoting cytokine and chemokine production, proliferation and survival [5]. CTLA-4 has a higher affinity for CD80 and CD86, which enables it to effectively compete with CD28 for these shared ligands [8, 9]. As the expression of CTLA-4 increases within the immune synapse, it attenuates CD28-mediated signaling, leading to the termination of T cell priming. Together these two opposing receptors modulate protective immune responses, while preventing overt autoimmune manifestations.

CTLA-4 is also expressed by activated populations of regulatory T (Treg) cells, which can impair productive anti-tumor immunity through a variety of suppressive mechanisms [10]. One such mechanism involves CTLA-4 on Treg cells mediating the physical removal of CD80 and CD86 from the surface of activated APCs by a process termed trans-endocytosis, which attenuates their stimulatory properties [11]. Consistent with this finding, several studies have demonstrated that the extracellular domain of CTLA-4 alone is sufficient for substantial T cell inhibitory function. For example, Treg cells expressing a membrane-anchored extracellular domain variant of CTLA-4, without the ability to transmit intracellular signaling, retained the ability to potently suppress T cell-mediated immune responses in mice [12]. However, the intracellular cytoplasmic region of CTLA-4 may contribute to the control of trafficking, level of expression, cellular location and timing of CTLA-4 appearance at the plasma membrane.

In preclinical mouse models, surrogate anti-CTLA-4 antagonist antibodies have shown compelling anti-tumor efficacy. Requisite to this tumoricidal activity was the ability of anti-mouse CTLA-4 antibodies to inhibit the interaction of CTLA-4 with CD80 and CD86 [13, 14]. Notably, optimal anti-tumor efficacy required the expression of mouse Fcγ receptors (FcγRs), in particular those of the activating subclass [15–17]. This mechanism was attributed to the interaction of CTLA-4-bound antibodies with FcγRs on the surface of effector cells within the tumor, such as macrophages and natural killer (NK) cells, and was correlated with the selective depletion of intratumoral Treg cells [15]. In addition to single agent activity, anti-mouse CTLA-4 antibodies have shown compelling anti-tumor efficacy in combination with other immunomodulatory antibodies, therapeutic vaccines and cell therapies, thereby supporting the versatility of neutralizing the CTLA-4 pathway to promote successful cancer immunotherapy [13].

Consistent with this preclinical evidence, clinical benefit has been observed with ipilimumab, a human IgG1 anti-CTLA-4 antibody, in patients with advanced stages of melanoma [18, 19]. The anti-tumor efficacy of anti-CTLA-4 antibodies in patients has been correlated with several predictive and pharmacodynamic (PD) biomarkers, including non-synonymous tumor mutational burden, changes in the TCR repertoire upon treatment, correlations of general patient immune status as well as increased frequencies of FcγRIIIA-expressing non-classical monocytes and reduced intratumoral Treg cell infiltration [18, 20–22]. Notably, ipilimumab has also shown remarkable combination efficacy with an anti-programmed cell death protein 1 (PD-1) antagonist antibodies [23]. Several clinical trials are underway with ipilimumab and a human IgG2 anti-CTLA-4 antibody, tremelimumab, alone and in combination with other immunomodulatory antibodies, tumor-targeting antibodies, small molecule therapies and therapeutic cancer vaccines [24, 25].

Here we sought to functionally interrogate the mechanisms of action for a novel human IgG1 anti-CTLA-4 antagonist antibody, AGEN1884, and to assess its activity and tolerability in a non-human primate model. Our studies show that AGEN1884 can potently modulate the immune synapse by antagonizing CTLA-4, which can be further potentiated in combination with other immunomodulatory antibodies. Our *in vitro* and *in vivo* assessments extended to a direct comparison of AGEN1884 with an IgG2 Fc variant, AGEN2041. Our findings uncover an FcγR-dependent mechanism that produced approximately a 40-fold difference in the pharmacological activity of an IgG1 versus IgG2 anti-CTLA-4 antagonist antibody. These findings may have clinical implications for antibodies targeting the CTLA-4 pathway, including consideration for pharmacokinetic (PK) and PD relationships. Our findings may also aid in the design and optimization of next generation anti-CTLA-4 antibodies and help guide their clinical application.

## Materials and methods

### Antibodies

AGEN1884 (anti-CTLA-4; IgG1), AGEN2041 (anti-CTLA-4; IgG2) and AGEN2034 (anti-PD-1; IgG4) were discovered using a proprietary mammalian display technology, Retrocyte Display™ [26]. The full sequences for each antibody are publicly available [27, 28]. Notably, the variable heavy (VH) and variable light (VL) chains for AGEN1884 and AGEN2041 are identical. IgG1, IgG2 or IgG4 variants of an antibody selective for the human cytomegalovirus (CMV) protein, glycoprotein B (clone SM5-1 (4Ab-028)), for which the sequence is also publicly available, were used as isotype control antibodies [29, 30]. The antibodies used for flow cytometry analysis of human peripheral blood mononuclear cell (PBMC) populations included: anti-CD3 (clone OKT3), CD4 (clone OKT4 or L200), CD8 (clone SK1), CD25 (clone BC96), CD28 (clone CD28.2), CTLA-4 (clone L3D10), interferon (IFN)-γ (clone B27) and tumor necrosis factor (TNF)-α (clone Mab11) purchased from BioLegend, and anti-Forkhead box P3 (FoxP3) (clone PCH101) purchased from Thermo Fisher Scientific.

### AGEN1884 binding to CTLA-4

A wildtype Jurkat cell line (ATCC; catalogue number TIB-152) and a Jurkat cell line engineered to express CTLA-4 constitutively on its surface (Promega; catalogue number CS186901B) were suspended in 2% fetal bovine serum (FBS) in phosphate-buffered saline (PBS). Both cell lines were used immediately after thawing and were not used beyond two passages. Cell authentication was performed by phenotyping for CD28 and CTLA-4 surface expression. Increasing concentrations of AGEN1884 or an isotype control (clone 4Ab-028) were added to CTLA-4-expressing or wildtype Jurkat cells and incubated for one hour at 4°C. Cell surface binding of AGEN1884 was detected using a phycoerythrin (PE)-labeled anti-human Ig secondary antibody (Fitzgerald Industries). Antibody binding was evaluated by flow cytometry based on the mean fluorescence intensity (MFI) of PE.

### Luminex ligand blocking assay

Recombinant CTLA-4-Fc coupled to microsphere beads (R&D Systems) was incubated with increasing concentrations of each antibody for one hour. Recombinant CD80-Fc or CD86-Fc (R&D Systems) labeled with PE (LYNX Rapid RPE Antibody Conjugation kit, Bio-Rad) were added. Binding of CD80-Fc or CD86-Fc was evaluated by Luminex based on the MFI of PE, normalized to the uninhibited signal without antibody and plotted as percent binding.

### Cell-based ligand blocking assay

CTLA-4 expressing CHO cells (GenScript; catalogue number M00530) were incubated for 30 min on ice with increasing concentrations of AGEN1884 or an isotype control. Cell authentication was performed by phenotyping for CTLA-4 surface expression. This cell line was used within six passages to ensure sustained surface expression. CD80-Fc and CD86-Fc fusion proteins were conjugated to Alexa Fluor 647 (AF647) using the AF647 labelling kit (Life Technologies), as per manufacturer’s instructions. Following the incubation of the cells, AF647-conjugated CD80-Fc or CD86-Fc fusion proteins were added directly to the cells at a final concentration of 0.625 μg/mL and incubated for 30 min on ice. Cells were washed and stained with Zombie Green viability dye (Biolegend). Binding of CD80-Fc or CD86-Fc was evaluated by flow cytometry based on the MFI of AF647.

### Epitope mapping

Epitope mapping of AGEN1884 was performed using hydrogen-deuterium exchange mass spectrometry (HDX-MS). The Fab fragment of AGEN1884 was prepared by direct expression. The extracellular domain (ECD) of human CTLA-4 was obtained as a His-tagged protein (AcroBiosystems). Size-exclusion chromatography (Tosoh TSK-gel super SW 3000 4.6 mm x 30 cm, 4 μm particle size, PBS as eluent at a flow rate of 0.35 mL/min) was used to confirm the formation of a complex in solution between the AGEN1884 Fab and the CTLA-4 ECD. Following denaturation and reduction of the ECD, peptide mapping of the CTLA-4 ECD was performed using on-column protease digestion with both pepsin and protease XIII. The resulting peptide mixture was analyzed by ultra-high pressure liquid chromatography-mass spectrometry (UHPLC-MS). Peptide MS-MS data were compared with the CTLA-4 sequence using Mascot software to identify specific peptides resulting in 99.2% coverage of the protein sequence. Comparative HDX-MS studies of the solutions of CTLA-4 ECD alone and in complex with AGEN1884 Fab were performed by diluting proteins into deuterium oxide labeling buffer followed by incubation for varying lengths of time (0, 60, 300, 1800, and 7200 sec). Deuterium exchange was quenched by addition of an equal volume of 4 M GnHCl/ 0.85 M TCEP buffer (pH 2.5). Resulting denatured and reduced samples were subjected to pepsin/protease XIII digestion and LC-MS analysis. Mass increases for individual peptides as compared to time zero indicated the level of deuterium incorporation. Significant decreases in the level of deuterium incorporation in specific ECD peptides between the ECD alone and in complex with AGEN1884 define the epitope of the antibody.

### Surface plasmon resonance

AGEN1884 was captured to a CM5 Biacore sensor chip surface using a human Fab capture system for analysis with a Biacore T200. Increasing concentrations of soluble recombinant CTLA-4 proteins from human (R&D Systems), cynomolgus macaques (Sino Biological) and mouse (Sino Biological) as well as human CD28 protein (Sino Biological) were assessed for binding to AGEN1884. Binding kinetic analyses were carried out using Biacore evaluation software (GE Healthcare version 3.0).

### CTLA-4 T cell reporter assay

One vial of thaw-and-use CTLA-4 overexpressing Jurkat cells genetically engineered with an IL-2-dependent luciferase reporter (Promega; catalogue number CS186901B) was thawed and resuspended in RPMI-1640 supplemented with 10% FBS, as per manufacturer's instructions. Increasing concentrations of AGEN1884 or an isotype control antibody were added to CTLA-4-expressing Jurkat cells with thaw-and-use Raji cells (Promega; catalogue number CS186901A). Both cell lines were used immediately after thawing without passaging. Cell authentication was performed by phenotyping for CD28 and CTLA-4 surface expression. After a ten-hour incubation at 37°C, an equal volume of Bio-Glo™ luciferase assay reagent (Promega) was added and luminescence was measured at an absorbance of 405 nm. The fold increase of relative light units in the presence of antibody compared to basal luciferase expression was plotted.

### Reference antibody sequences

The commercial reference antibodies, nivolumab (Bristol-Myers Squibb) and pembrolizumab (Merck), used in the Staphylococcal enterotoxin A (SEA) peptide assay in combination with AGEN1884 were both purchased from Myoderm (Norristown, PA). The two non-commercial reference antibodies used in the SEA peptide assay in combination with AGEN1884 include the IgG4 anti-CD137 antibody (clone 10C7) and the IgG4 anti-lymphocyte-activation gene 3 (LAG-3) antibody (clone 25F7), for which both sequences are publicly available [31, 32]. The variable heavy chain domain (VH) and variable light chain domain (VL) of 10C7 consist of: QVQLQQWGAGLLKPSETLSLTCAVYGGSFSGYYWSWIRQSPEKGLEWIGEINHGGYVTYNPSLESRVTISVDTSKNQFSLKLSSVTAADTAVYYCARDYGPGNYDWYFDLWGRGTLVTVSS and EIVLTQSPATLSLSPGERATLSCRASQSVSSYLAWYQQKPGQAPRLLIYDASNRATGIPARFSGSGSGTDFTLTISSLEPEDFAVYYCQQRSNWPPALTFGGGTKVEIK, respectively [32]. The VH and VL domain of 25F7 consist of: QVQLQQWGAGLLKPSETLSLTCAVYGGSFSDYYWNWIRQPPGKGLEWIGEINHNGNTNSNPSLKSRVTLSLDTSKNQFSLKLRSVTAADTAVYYCAFGYSDYEYNWFDPWGQGTLVTVSS and EIVLTQSPATLSLSPGERATLSCRASQSISSYLAWYQQKPGQAPRLLIYDASNRATGIPARFSGSGSGTDFTLTISSLEPEDFAVYYCQQRSNWPLTFGQGTNLEIK, respectively [31].

### Primary antigen-presenting cell and T cell co-culture assay

Human blood was purchased from Research Blood Components (Boston, MA). Human PBMC were isolated by density centrifugation and stimulated with SEA peptide (Toxin Technology), which mediates interactions between MHC class II molecules and CD86 on APCs and the TCR and CD28 on T cells, respectively [33, 34]. AGEN1884 or AGEN2041 were added alone or in combination with anti-PD-1 (nivolumab, pembrolizumab or AGEN2034), LAG-3 (clone 25F7), CD137 (clone 10C7) or an isotype control antibody (clone 4Ab-028). After 5 days, co-culture supernatants were collected and interleukin-2 (IL-2) was measured as an endpoint of enhanced T cell responsiveness using an AlphaLISA® immunoassay kit (Perkin-Elmer), as per manufacturer’s instructions.

### Fcγ receptor reporter assay

CTLA-4-expressing Jurkat cells (Promega; catalogue number CS186901B) were incubated with increasing concentrations of AGEN1884 or an isotype control antibody. Effector Jurkat cells engineered to express FcγRIIIA_V158_, FcγRIIIA_F158_, or FcγRIIA_H131_ with an NFAT-dependent firefly luciferase reporter gene (Promega; catalogue numbers G7102, CS1324F08 and CS178101, respectively) were suspended in RPMI-1640 supplemented with 10% low-IgG FBS (Sigma) and added at an effector-to-target cell ratio of 2.5:1. All effector Jurkat cell lines were used within six passages. Cell authentication was performed by phenotyping for CD28 and FcγRIIIA (clone 3G8) or FcγRIIA (clone IV.3) surface expression. After a 20-hour incubation at 37°C, an equal volume of Bio-Glo™ luciferase assay reagent was added to each well and luminescence was measured at an absorbance of 405 nm.

### Fcγ receptor binding

CHO cell lines genetically engineered to express different FcγRs (FcγRI, FcγRIIA (131R variant) or FcγRIIIA (158V or 158F variants)) (Collection de Cultures de Microorganismes, Institut Pasteur; catalogue numbers CNCM I-4383, CNCM I-4385, CNCM I-4389, CNCM I-4388, respectively). A Jurkat cell line genetically engineered to express FcγRIIA 131H variant was also used (Promega; catalogue number CS178101). All cell lines were used within six passages. Cell authentication was performed by phenotyping for FcγRI (clone 10.1), FcγRIIB (clone 2B6), FcγRIIA (clone IV.3) and FcγRIIIA (clone 3G8) surface expression. All cell lines were incubated with increasing concentrations of AGEN1884 or AGEN2041. Following a one-hour incubation, binding of the antibodies was detected using a PE-conjugated goat F(ab’)2 fragment specific for anti-human IgG (Jackson ImmunoResearch). After a 30-min incubation, the cells were fixed with 1% paraformaldehyde and antibody binding was evaluated by flow cytometry based on the MFI of PE.

### Antibody-dependent cellular cytotoxicity assay with NK-92 effector cells

CTLA-4-expressing Jurkat cells (Promega) were co-cultured with a FcγRIIIA (158V variant)-expressing NK-92 cell line (Genscript) at an effector-to-target cell ratio of 5:1 in the presence of increasing concentrations of AGEN1884 or an isotype control antibody. This cell line and assay were developed by GenScript. Following a six-hour incubation, the supernatants were collected and measured for lactate dehydrogenase (LDH) release at an absorbance of 492 nm using a Cytotoxicity Detection Kit (Roche Applied Science), as per manufacturer’s instructions. The percent of target cell-specific lysis was calculated as: (optical density (OD) _Sample data_—OD _Target cells plus effector cells_) / (OD _Maximum lysis_—OD _Minimum lysis_) * 100. The maximum lysis was achieved using target cells with 1% Triton X-100 solution and the minimum lysis was determined from target cells alone.

### Antibody-dependent cellular cytotoxicity assay with primary effector cells

Human blood was purchased from Research Blood Components (Boston, MA). To generate the target cell populations, Treg or effector T (Teff) cells were purified from isolated PBMC using the human Regulatory T Cell Isolation Kit (Miltenyi Biotec) or Pan T Cell Isolation Kit (Miltenyi Biotec), respectively, as per manufacturer’s instructions. Treg or Teff cells were then cultured in RPMI-1640 supplemented with 10% FBS and 500 U/mL of human IL-2 (R&D Systems) with beads coated with anti-CD3 and CD28 antibodies (Miltenyi Biotec) for seven days at 37°C. Target cell expression of cell surface CTLA-4 was confirmed using fluorochrome-conjugated antibodies targeting CD3, CD4, CD8, CD25 and CTLA-4 and cells were stained in PBS containing 2% FBS. T cells were subsequently fixed and permeabilized using the FoxP3 Transcription Factor Staining Buffer kit (Thermo Fisher Scientific), as per manufacturer’s instructions, stained for intracellular FoxP3 expression and analyzed by flow cytometry.

Following confirmation of CTLA-4 expression, activated Teff and Treg cells were co-cultured with isolated primary NK cells using the human NK Cell Isolation Kit (Miltenyi Biotec) at a 5:1 effector-to-target cell ratio. AGEN1884, AGEN2041 or an isotype control antibody (clone 4Ab-028) were added at increasing concentrations and incubated for 4 hours at 37°C. Target cell-specific lysis was assessed by flow cytometry and calculated as the percentage of CD3-positive target cells that also stained positive with the viability dye, 7-Aminoactinomycin D (7-AAD) (BD Biosciences), minus the background lysis observed with no antibody.

### Enzyme-linked immunoSpot assay

Multiscreen ELISpot plates (EMD Millipore) were pre-wetted with 35% ethanol and washed with PBS. Plates were coated with anti-IFN-γ antibody solution (Mabtech) at 15 μg/mL overnight at 4°C and blocked with RPMI-1640 supplemented with 10% FBS for 2 hrs at 37°C. Hepatitis B surface antigen (HBsAg) at a final concentration of 5 μg/mL and PBMC resuspended in RPMI-1640 supplemented with 10% FBS were added. Phytohaemagglutinin (PHA) at a final concentration of 2 μg/mL was used as a positive control. The plate was incubated for 40 hrs at 37°C. The plate was washed with PBS supplemented with 0.05% Tween-20, and 1 μg/mL of anti-IFN-γ biotinylated antibody (Mabtech) in PBS with 1% BSA was added and incubated for two hours at 37°C. The plate was washed six times with PBS supplemented with 0.05% Tween-20 and streptavidin-horseradish peroxidase (HRP) was added and incubated for one hour. During this hour, one 3-Amino-9-ethylcarbazole tablet was dissolved in 2.5 mL of N,N-dimethylformamide and added to 47.5 mL of 7.4% 0.1 N glacial acetic acid, 17.6% 0.1 N sodium acetate and 75% distilled, deionized H_2_O solution supplemented with 50 μL of 30% hydrogen peroxide. This final substrate solution was added to the plate and incubated for three minutes. The plate was thoroughly washed with distilled water to stop the development reaction, dried overnight and read using the S6 Macro analyzer (ImmunoSpot).

### Cytokine release assay

Undiluted fresh blood (used within three hours of time of blood draw) from ten healthy control donors (Research Blood Components) was incubated with either soluble (aqueous phase) AGEN1884 (0.1, 1, 10 or 100 μg/mL) or plate-bound AGEN1884 (0.002, 0.02, 0.2 or 2 μg/well) at 37°C for 24 hrs. As controls, whole blood samples from the same donors were incubated with identical concentrations of two comparator biologics, cetuximab and alemtuzumab (Myoderm) [35]. As additional controls, whole blood from each donor was incubated with PBS (negative control) or Staphylococcal enterotoxin B (SEB; ProImmune) (positive control). Following the incubation, the plasma was analyzed for the presence of IL-2, IL-4, IL-6, IL-8, IL-10, IFN-γ and TNF-α using a protein microarray format.

### Agonistic potential of AGEN1884 on purified human T cells

Human PBMC were isolated from whole blood (Research Blood Components) by density centrifugation. Thereafter, negative magnetic bead T cell isolation (Miltenyi Biotec) was performed and purified T cells were rested overnight at 37°C in RPMI-1640 supplemented with 10% FBS. Anti-CD3 antibody (clone SP34; 5 μg/mL) and increasing doses of AGEN1884 or an isotype control were pre-coated onto a plate and T cells were added and incubated for four days at 37°C. At the end of the incubation, culture supernatants were harvested and Th1/Th2 cytokine levels (IFN-γ, IL-10, IL-12p70, IL-13, IL-1β, IL-2, IL-4, IL-5, IL-8 and TNF-α) were measured using the human Th1/Th2 10-Plex Meso Scale Discovery kit (MSD), as per manufacturer’s instructions. Furthermore, cell pellets were collected and incubated with Golgi Plug™ (BD Biosciences) for six hours at 37°C. After the incubation, cells were stained with the viability dye, Near-IR amine dye (Life Technologies), followed by anti-CD4 and CD8 surface T cell lineage markers. The cells were then permeabilized with Cytofix/Cytoperm™ (BD Biosciences) for detection of intracellular production of IFN-γ and TNF-α.

### Non-human primate vaccine model

Cynomolgus macaques (*Macaca fascicularis*) of Chinese origin were sourced from a contract laboratory breeding facility in Texas. They were housed in standard size stainless steel cages equipped with a stainless steel mesh floor and an automatic watering valve. Primary enclosures were as specified in the USDA Animal Welfare Act (9 CFR, Parts 1, 2, and 3) and as described in the *Guide for the Care and Use of Laboratory Animals*. Unless precluded for technical, scientific or health reasons, animals were socialized to provide for psychological enrichment, with the exception of times when they were separated for designated study procedures/activities. Up to three animals of the same sex were commingled within dose groups after initial compatibility tests were completed. Animals were provided certified primate chow (25% protein) daily in amounts appropriate for the size and age of the animal. In addition, the diet was supplemented with fruit or vegetables at least 2–3 times weekly. Water was provided *ad libitum* and was regularly tested to ensure there were no known contaminants. Veterinary care was available throughout the course of the study and animals were observed at least once daily. Scheduled euthanasia was conducted by an intravenous (IV) injection of ketamine followed by Beuthanasia® and exsanguination. This research was approved by the Institutional Animal Care and Use Committee (IACUC) from Charles River Laboratories (Reno, NV, USA) with the study numbers 20091078 and 464501. On days 1 and 29, 10 mg/kg of AGEN1884 or a vehicle solution were administered to the appropriate group of animals *via* IV injection (slow bolus) into the saphenous vein. Keyhole limpet hemocyanin (KLH; 750 μg) (Thermo Fisher Scientific) was administered intramuscularly, and 30 μg of Hepatitis B virus surface antigen (HBsAg) vaccine (ENGERIX-B^®^) (GlaxoSmithKline) was administered in three subcutaneous injections. Serum was collected on days 1, 15, 29, 43, 57 and 69. Anti-HBsAg (IgG) and anti-KLH antibody (IgM and IgG) titers were measured at each timepoint using a validated ELISA, as previously described [36].

In a second study, the anti-PD-1 antibody, nivolumab (Opdivo®), at 3 mg/kg was administered by IV infusion into the saphenous vein. The indwelling catheter was then flushed with saline and a second infusion was conducted shortly after with AGEN1884 (10 mg/kg). Nivolumab and AGEN1884 were each infused over approximately 10–15 minutes. After the final dose administration, a 1 mL flush of sterile saline was administered. Blood was collected prior to dosing on days -14 and -7, on the day of dosing and at days 2, 3, 7, 10, 14, 18, 22, 26 and 30 post dosing for subsequent analyses.

### Immunophenotyping of PBMC

Cynomolgus PBMC were stained with Near-IR amine dye (Life Technologies) to measure viability. The cells were washed with 2% FBS in PBS and blocked with TruStain FcX™ Fc Receptor blocking solution (BioLegend) for 15 min at 4°C. The cells were stained with an antibody cocktail containing non-human primate cross-reactive antibodies specific for CD4 (clone L200), CD8 (clone SK1), CD3 (clone SP34.2), CD20 (clone 2H7), CD16 (clone VEP13), HLA-DR (clone L243), CD38 (clone AT-1), PD-1 (clone EH12.1), CD25 (clone 4E3), CD28 (clone 28.8) and CD95 (clone DX95). Cells were fixed/permeabilized with Cytofix/Cytoperm buffer (BD Biosciences), as per manufacturer’s instructions. After washing, anti-Ki67 (clone B56) antibody was added and incubated for 15 min at 4°C. Cells were washed again with 2% FBS in PBS and the expression of Ki67 was evaluated by flow cytometry.

### Pharmacokinetic determination for AGEN1884 in cynomolgus macaques

AGEN1884 plasma concentrations from cynomolgus macaques were measured using a validated ELISA method. This method utilized a traditional ligand binding assay format using human CTLA-4 as a capture reagent. Samples and AGEN1884 standards and controls were added followed by detection with a monkey-adsorbed goat anti-human IgG-HRP conjugate (BioAgilytix). Lastly, a tetramethylbenzidine (TMB) chromogenic substrate was added, the reaction was stopped with a 2N H_2_SO_4_ solution and the plate was read at 450 nm.

### Cytokine profile

Serum cytokine responses were evaluated with a non-human primate Meso Scale Diagnostic V-plex cytokine array kit (MSD), as per manufacturer’s instructions. Serum samples were collected pre-dose (0 hrs) and 2, 6 and 24 hrs post-infusion from days 1 and 29. The following panel of cytokines, chemokines and pro-inflammatory factors were evaluated: IL-1β, IL-2, IL-6, IL-7, IL-8, IL-10, IFN-γ and TNF-β. Cynomolgus macaque serum samples collected longitudinally during the study were frozen down for batch processing and analysis.

## Results

### AGEN1884 potently and selectively antagonizes the CTLA-4 pathway

**Fig 1:**
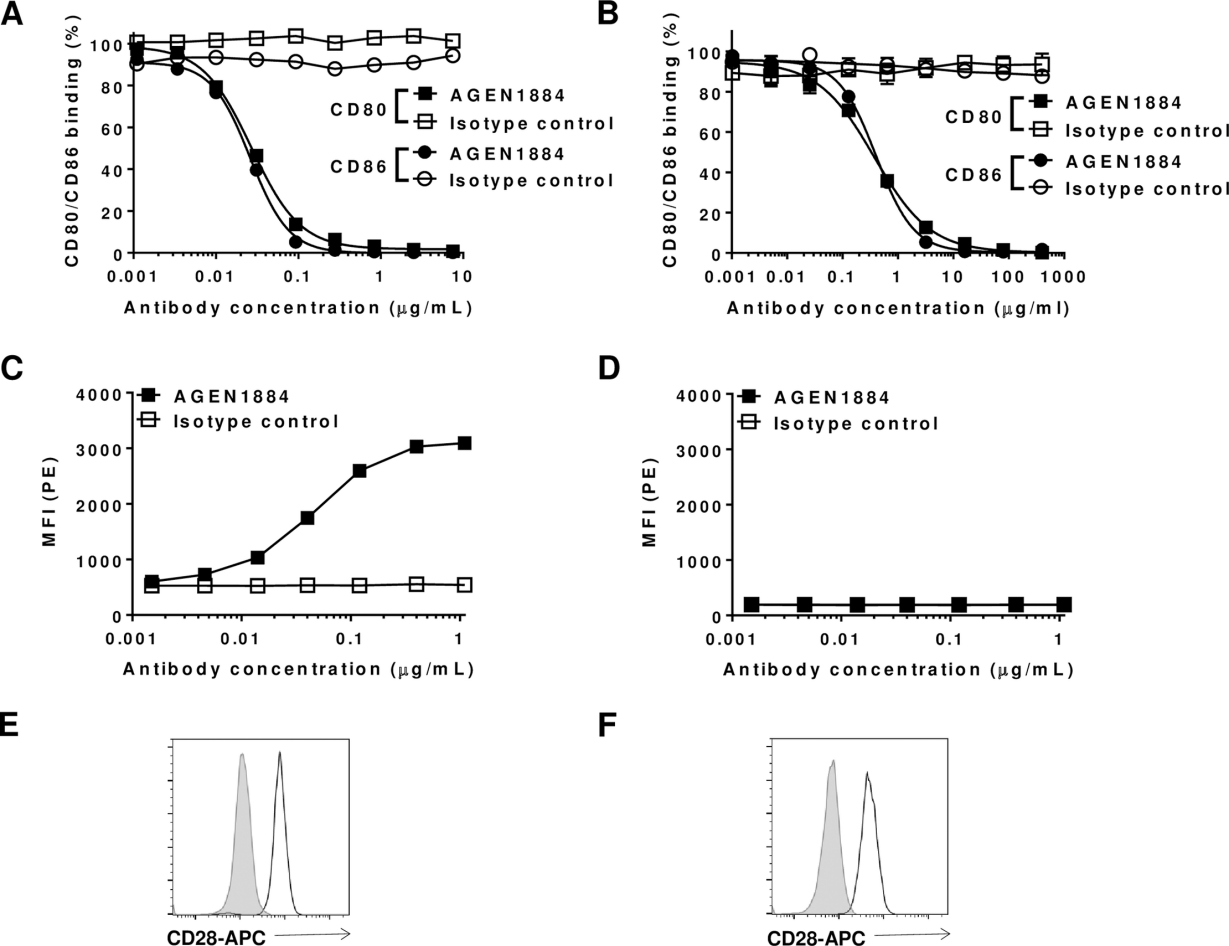
AGEN1884 binds CTLA-4 and blocks CTLA-4 from interacting with CD80 and CD86. (A) Binding of fluorescently-labeled CD80-Fc or CD86-Fc (1 nM) in the presence of increasing concentrations of AGEN1884 or an IgG1 isotype control. Binding to CTLA-4-linked microspheres was assessed using Luminex. (B) CTLA-4-expressing CHO cells were pre-incubated with increasing concentrations of AGEN1884 or an IgG1 isotype control followed by the addition of a fixed concentration of fluorescently-labeled CD80-Fc or CD86-Fc (0.625 μg/ml). Binding of CD80-Fc or CD86-Fc to the CHO cells was assessed by flow cytometry. (C-D) AGEN1884 binding to a (C) Jurkat cell line genetically engineered to express human CTLA-4 or (D) wildtype (CTLA-4-negative) Jurkat cell line. Expression of CD28 was also assessed using an anti-CD28 antibody (empty histogram) compared to an isotype control (filled histogram) on the (E) CTLA-4-expressing and (F) wildtype (CTLA-4-negative) cells lines. (A-D) Representative data from one of three experiments indicate the mean ± SEM in each treatment group.

AGEN1884 was selected from a panel of candidate antibodies based on its ability to block the interaction of recombinant CTLA-4 with CD80 and CD86 (Fig 1A, S1 Fig). Similarly, AGEN1884 was confirmed to potently antagonize CD80 and CD86 binding to cell-expressed CTLA-4 (Fig 1B). Requisite to its consideration as a development candidate, AGEN1884 was confirmed to cross-react with cynomolgus macaque CTLA-4 (S1 Table). Indicative of its selectivity for CTLA-4, AGEN1884 did not bind to other CD28-related family members including CD28, PD-1, inducible co-stimulator (ICOS) or B- and T-lymphocyte attenuator (BTLA) (S2A and S2B Fig). While the overall sequence homology between CTLA-4 and CD28 is approximately 20% (31% identity at the amino acid level), both receptors share the MYPPPY motif that is essential for binding to their shared ligands, CD80 and CD86 [37]. Therefore, the binding selectivity of AGEN1884 for CTLA-4 versus CD28 was further substantiated using two additional orthogonal methods: 1) flow cytometry to evaluate binding of a dose-titration of AGEN1884 on a T cell line expressing CD28 or the same cell line engineered to co-express CTLA-4 in addition to CD28; and 2) by surface plasmon resonance (SPR) to compare the binding of AGEN1884 to CTLA-4 versus CD28 (Fig 1C–1F, S2C and S2D Fig). Under both assay conditions AGEN1884 selectively recognized CTLA-4, with no cross-reactivity detected to CD28.

### AGEN1884 binds to an epitope on human CTLA-4 that is consistent with ligand blockade

**Fig 2:**
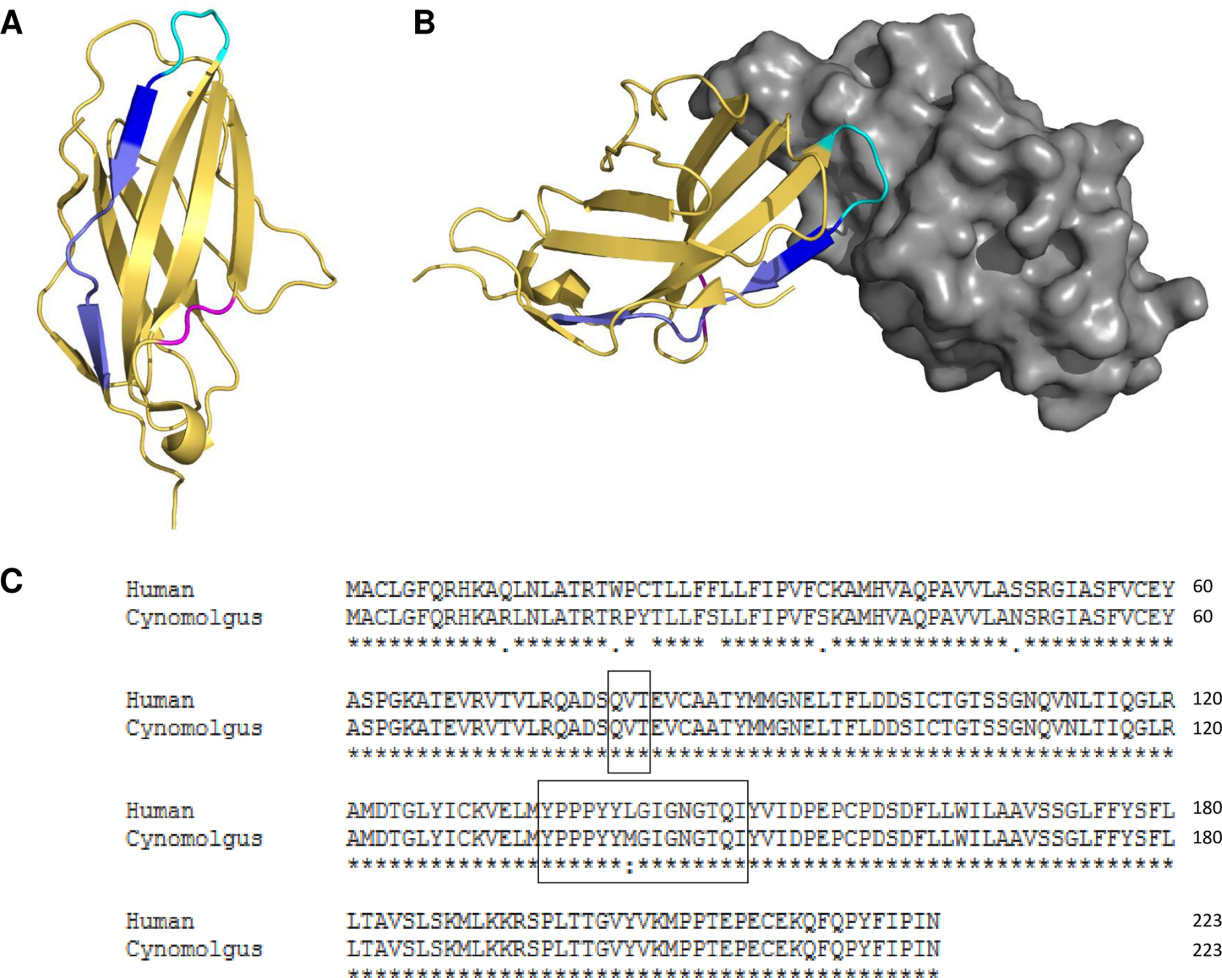
AGEN1884 epitope. (A) Mapping by hydrogen-deuterium exchange mass spectrometry (HDX-MS) on the CTLA-4 structure (PDB 1I8L). Ribbon representation of CTLA-4 highlighting residues identified as having reduced HDX by HDX-MS: residues 80–82 (QVT, magenta), 135–139 (YPPPY, cyan), 140–141 (YL, dark blue), 142–149 (GIGNGTQI, pale blue). (B) Structure of the human co-stimulatory complex CD80 (grey)/CTLA-4 (yellow) (PDB 1I8L). Residues having reduced HDX are indicated as in panel (A). The view of the structure highlights the loop region comprised of residues at positions 135–149, which encompasses a turn-loop that directly interacts with CD80. (C) Sequence alignment between human and cynomolgus macaque CTLA-4. An asterisk indicates identical residues, a colon indicates conservation between groups of strongly similar properties and a period indicates conservation between groups of weakly similar properties. Solid line boxes indicate residues identified as having reduced HDX.

To define the molecular epitope of AGEN1884 on human CTLA-4, HDX-MS was performed using the recombinant ECD of CTLA-4 complexed with the antigen-binding fragment of AGEN1884. Differences in the rate and extent of HDX were used to define the amino acid residues in CTLA-4 protected (i.e. bound) by AGEN1884. HDX-MS identified a significant reduction in deuterium uptake at amino acids 80–82 and 135–149 of CTLA-4, with key binding residues identified at tyrosine position 140 (Y140) and leucine at position 141 (L141) (Fig 2A and 2B). Interestingly, this epitope includes residues 135–139 (YPPPY), which is part of the MYPPPY motif of CTLA-4 and CD28 [38–41]. Residues 135–139 and adjacent Y140 and L141 form an extended loop-turn structure in CTLA-4 that is the main interface with receptor CD80 as determined by X-ray crystallography [41]. Together, these data demonstrate that the CTLA-4 epitope bound by AGEN1884 overlaps considerably with the binding site for its natural ligands, CD80 and CD86. In addition, the CTLA-4 epitope recognized by AGEN1884 is highly conserved between humans and cynomolgus macaques, which is consistent with its cross-reactivity profile to this non-human primate species (Fig 2C).

### AGEN1884 effectively modulates the immune synapse and cooperates with other immunomodulatory antibodies to enhance T cell responsiveness

**Fig 3:**
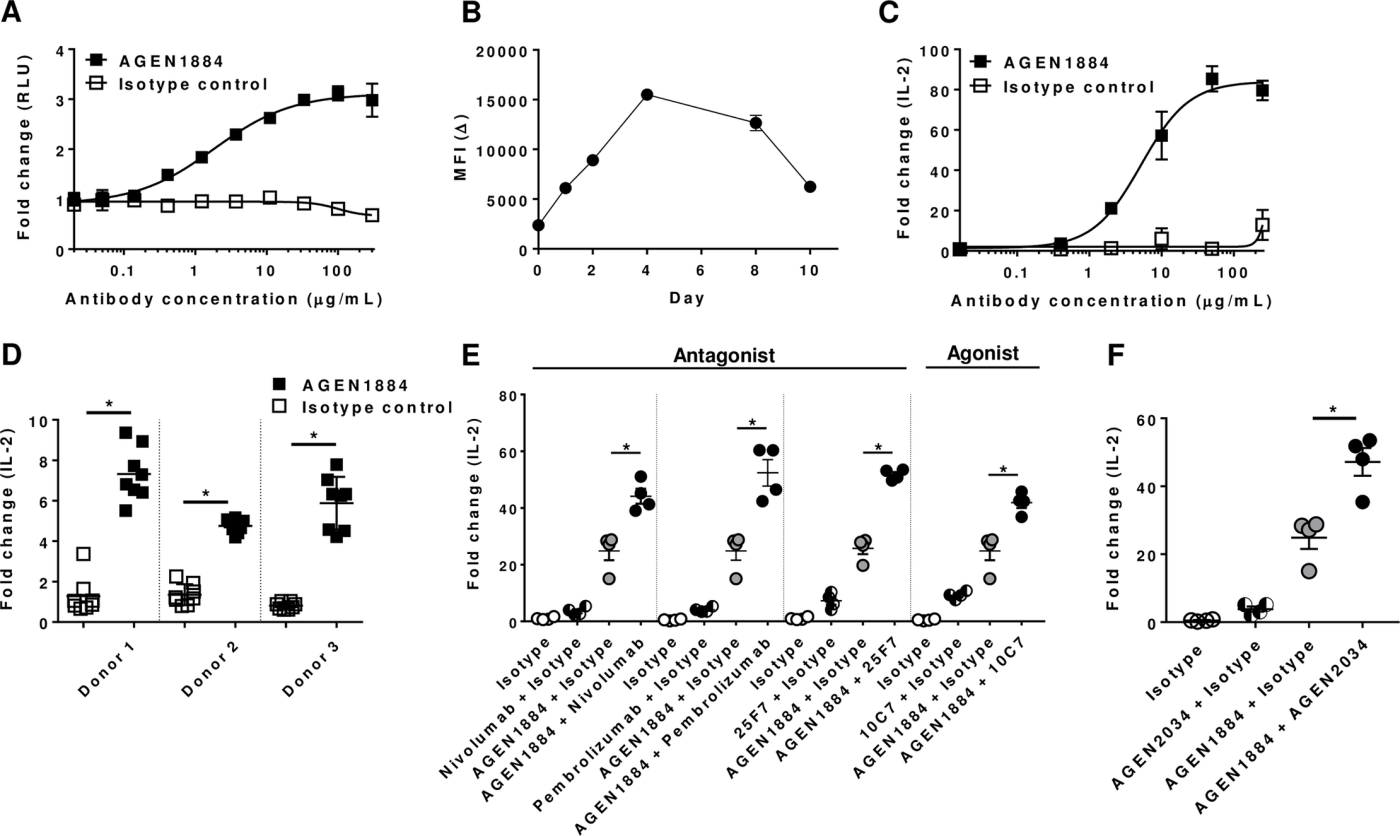
AGEN1884 increases T cell activation alone and in combination with other immunomodulatory antibodies. (A) A Jurkat T cell line genetically engineered to express CTLA-4 with an IL-2-dependent luciferase reporter was co-cultured with CD80/CD86-positive artificial APCs in the presence of increasing concentrations of AGEN1884 or an isotype control antibody. The fold increase in relative light units (RLU) relative to baseline is shown. (B) CTLA-4 expression was measured on T cells on days 1, 2, 4, 8 and 10 following stimulation with a sub-maximal concentration of SEA peptide (100 ng/mL). Primary human PBMC were stimulated with a sub-maximal concentration of the SEA peptide (100 ng/mL) and (C) increasing doses of AGEN1884, (D) single dose of AGEN1884 (10 μg/mL), (E) AGEN1884 (10 mg/mL) ± nivolumab (anti-PD-1 antagonist antibody), pembrolizumab (anti-PD-1 antagonist antibody), 25F7 (anti-LAG-3 antagonist antibody), 10C7 (anti-CD137 agonist antibody) (10 mg/mL) or (F) AGEN1884 (10 mg/mL) ± AGEN2034 (anti-PD-1 antagonist antibody) (10 mg/mL). Replicate cell supernatants were collected after 5 days for measurement of IL-2. Representative data from one of three experiments indicate the mean ± SEM in each treatment group (n = 3). (D-F) Data were analyzed using a Student’s t-test. Significant differences depicted were p<0.05 (*).

The functional outcome of AGEN1884-mediated blockade of the CTLA-4 pathway during T cell activation was assessed using a TCR reporter cell assay. Briefly, a human T cell line was engineered to constitutively express CTLA-4 and a luciferase reporter gene downstream of an IL-2 promoter that could be triggered *via* TCR and CD28 co-activation. Reporter T cells were co-cultured with APCs expressing CD80 and CD86 together with an artificial T cell activator targeting CD3 (anti-CD3 scFv). In the absence of AGEN1884, CTLA-4-mediated sequestration of CD80 and CD86 impaired reporter activity. By contrast, the addition of increasing concentrations of AGEN1884 led to a dose-dependent increase in IL-2-driven reporter gene activation, mediated by CD80 and CD86 binding to and activating CD28 (Fig 3A).

Next, to evaluate the functional impact of AGEN1884 on primary human T cell responses, PBMC were stimulated with SEA peptide together with a dose titration of AGEN1884. In this assay, the SEA peptide mediates the interactions between MHC class II molecules on APCs and a subset of specific Vβ regions of the TCR repertoire in addition to CD28 and CD86 co-receptor engagement [33, 34]. IL-2 secretion was measured as an endpoint of enhanced T cell responsiveness, which is indicative of improved TCR signaling and NFAT activation [42]. During the co-culture assay, CTLA-4 expression continued to increase from day one to four post SEA peptide stimulation (Fig 3B). In the presence of increasing concentrations of AGEN1884, there was a dose-dependent increase of IL-2 secretion into the culture supernatant, as compared to an isotype control antibody (Fig 3C). Although the range of IL-2 responses varied among donors (200–2000 pg/mL), augmented T cell IL-2 production in the presence of AGEN1884 was consistently observed from PBMC isolated from multiple healthy donors (Fig 3D).

Importantly, CTLA-4 forward signaling has been reported to directly suppress proximal TCR signaling through inhibition of zeta-chain associated protein kinase-70 (ZAP-70) phosphorylation, mediated by reduced binding to the lymphocyte-specific protein tyrosine (Lck) kinase [43]. To exclude the possibility that AGEN1884 might mediate CTLA-4 forward signaling, purified primary human T cells were incubated with an anti-CD3 antibody to activate proximal TCR signaling events. AGEN1884 or an IgG1 isotype control antibody were included as either soluble or plate-bound (complexed) formats. After four days, CD8^+^ T cells were evaluated for production of intracellular IFN-γ and TNF-α as well as secreted IL-10, IL-12p70, IL-13, IL-1β, IL-2, IL-4, IL-5 and IL-8. Increasing concentrations of either soluble or plate-bound AGEN1884 had no impact on the level of these inflammatory cytokines, including IFN-γ produced by TCR stimulated CD8^+^ T cells, as compared to an isotype control antibody (S3A and S3B Fig).

Antagonist antibodies to CTLA-4 are in clinical trials to evaluate their anti-tumor efficacy alone and in combination with other therapeutic antibodies, including those targeting the PD-1 and PD-L1 signaling axis [44]. To determine if AGEN1884 cooperated with blockade of the PD-1 pathway, SEA peptide-stimulated PBMC were incubated with AGEN1884 alone or in combination with two commercially available anti-PD-1 antibodies, nivolumab or pembrolizumab (Fig 3E). AGEN1884 exhibited enhanced T cell responsiveness in combination with both anti-PD-1 antibodies. This cooperativity was further corroborated with a novel anti-PD-1 antibody, AGEN2034 (Fig 3F). Like other PD-1 antagonists, AGEN2034 potently inhibited the PD-1 pathway by blocking ligand engagement and showed single agent activity in a primary T cell assay (S4A–S4D Fig). Taken together, these findings are consistent with previous reports showing that co-blockade of the CTLA-4 and PD-1/PD-L1 pathways may afford superior pharmacological modulation of antigen-specific T cells over single therapeutic agents [44, 45].

In addition to T cell responsiveness to AGEN1884 in combination with antibodies targeting the PD-1 pathway, we sought to determine if AGEN1884 would combine with other antagonist or agonist antibodies targeting immune checkpoint receptors. Here we tested AGEN1884 in combination with an anti-LAG-3 antagonist antibody (clone 25F7) or an anti-CD137 agonist antibody (clone 10C7) [31, 46]. AGEN1884 combined effectively with anti-LAG-3 or anti-CD137 antibodies to significantly enhance T cell responsiveness, as compared to either single agent alone (Fig 3E). The cooperation of AGEN1884 with antibodies targeting both co-inhibitory and co-stimulatory receptors establishes the potential versatility of AGEN1884 in combination regimens in the clinic.

### AGEN1884 engages Fcγ receptors and has the potential to mediate antibody-dependent cellular cytotoxicity toward activated regulatory T cells

**Fig 4:**
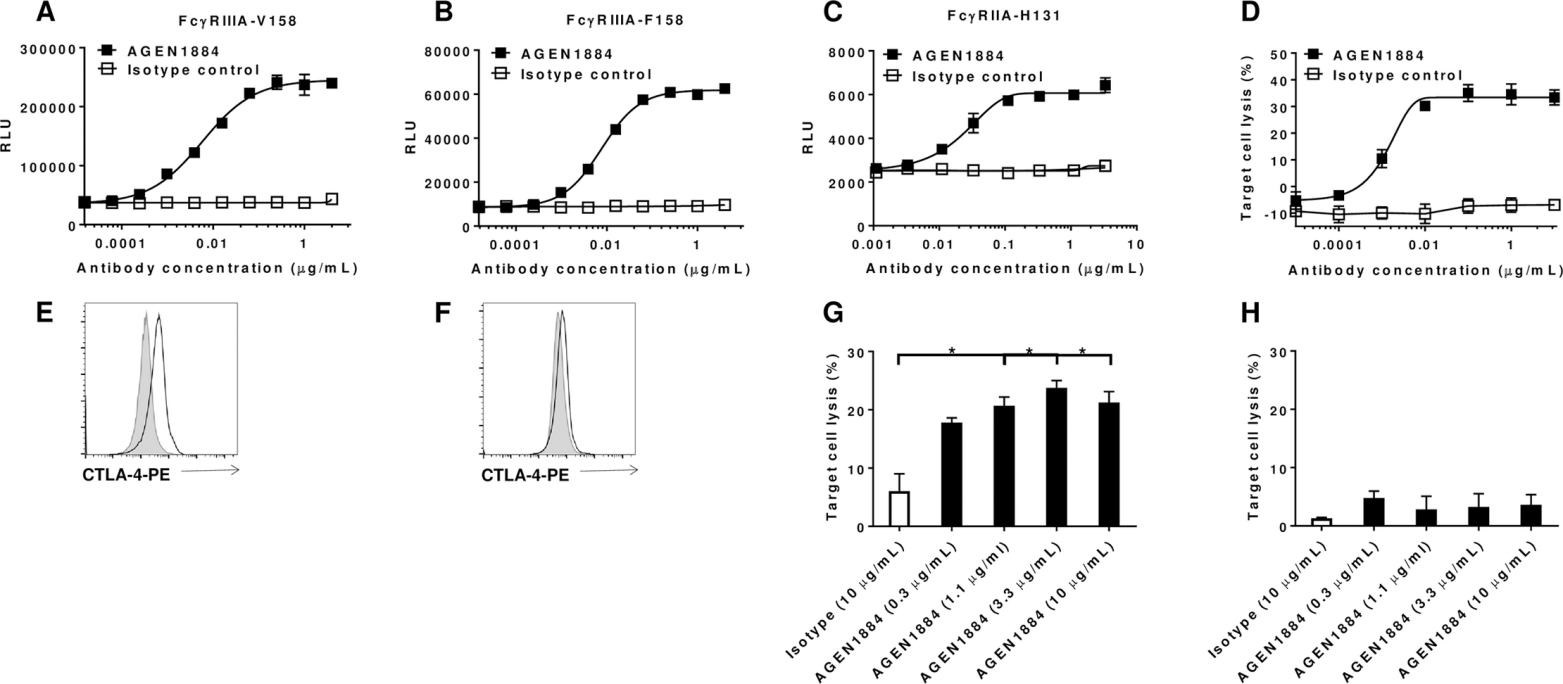
Fcγ receptor signaling and antibody-dependent cellular cytotoxicity of CTLA-4-expressing cells with AGEN1884. (A-C) Jurkat cell lines genetically engineered to express FcγRs upstream of a NFAT-dependent luciferase reporter were co-cultured with CTLA-4-expressing cells and increasing doses of AGEN1884 or an isotype control. Signaling through (A) FcγRIIIA-V158-, (B) FcγRIIIA-F158-, or (C) FcγRIIA-H131-expressing cells was assessed based upon luciferase expression, which is shown as relative light units (RLU). (D) The cytotoxicity of CTLA-4^high^ Jurkat cells by CD16-expressing NK-92 cells based on lactate dehydrogenase (LDH) release in the presence of increasing concentrations of AGEN1884 or an isotype control antibody. (E-F) Extracellular CTLA-4 expression (empty histogram) compared to the isotype control (filled histogram) was measured on (E) CD3^+^FoxP3^+^ or (F) CD3^+^FoxP3^-^ cells. The lysis of (G) CTLA-4^high^CD3^+^FoxP3^+^ target cells or (H) CTLA-4^low^CD3^+^FoxP3^-^ target cells by primary NK cells in the presence of increasing concentrations of AGEN1884 compared to an isotype control. Representative data from one of three experiments indicate the mean ± SEM in each treatment group (n = 2–3). (G, H) Data were analyzed using a Student’s t-test for each dose of AGEN1884 compared to the isotype. Significant differences depicted were p<0.05 (*).

Preclinical studies in mice have shown that optimal anti-tumor activity of anti-CTLA-4 antagonist antibodies required co-engagement of FcγRs expressed by tumor-associated effector cells [15–17, 47]. The mechanistic explanation for this finding is that anti-CTLA-4 antibodies with an Fc region that engages activating FcγRs are able to selectively deplete CTLA-4-expressing intratumoral Treg cells *via* effector cell activity [48]. As a glycosylated human IgG1 antibody, AGEN1884 co-engages activating and inhibitory FcγRs (S5 Fig). To determine if activating FcγR binding corresponded with downstream FcγR signaling, we compared the ability of AGEN1884, when bound to CTLA-4-expressing target cells, to signal through activating FcγRIIA and FcγRIIIA. These activating FcγRs have been implicated in antibody-mediated effector functions, including ADCC or antibody-dependent cellular phagocytosis (ADCP) [49]. ADCC is a mechanism by which antibody-opsonized cells are depleted by FcγRIIIA-expressing effector cells, such as NK cells or myeloid cell populations [50]. By contrast, ADCP describes the phagocytosis of antibody-opsonized cells by effector cells expressing FcγRIIA and/or FcγRIIIA, such as monocytes and macrophages [51]. AGEN1884 effectively engaged and signaled through both activating FcγRs (Fig 4A–4C). Moreover, in the presence of a FcγRIIIA-expressing NK cell line (NK-92), AGEN1884 induced ADCC of CTLA-4-expressing target cells (Fig 4D).

The ability of AGEN1884 to mediate ADCC against activated primary Treg and Teff cells expressing CTLA-4 was evaluated using primary NK cells. Briefly, primary Treg or Teff cells were isolated and activated to upregulate cell surface expression of CTLA-4 (Fig 4E and 4F). Higher cell surface expression of CTLA-4 was consistently observed on Treg compared to Teff cells following activation (Fig 4E and 4F). Activated Treg or Teff cells co-cultured with NK cells in the presence of AGEN1884 showed dose-dependent ADCC against high CTLA-4-expressing Treg cells, but minimal activity toward activated Teff cells (Fig 4G and 4H). Therefore, AGEN1884, by virtue of its IgG1 Fc region, has the ability to bind to CTLA-4 on Treg cells within the tumor and co-engage FcγRs on effector cells to mediate their selective depletion.

### Pharmacokinetics, tolerability and immunogenicity of AGEN1884 in cynomolgus macaques

**Fig 5:**
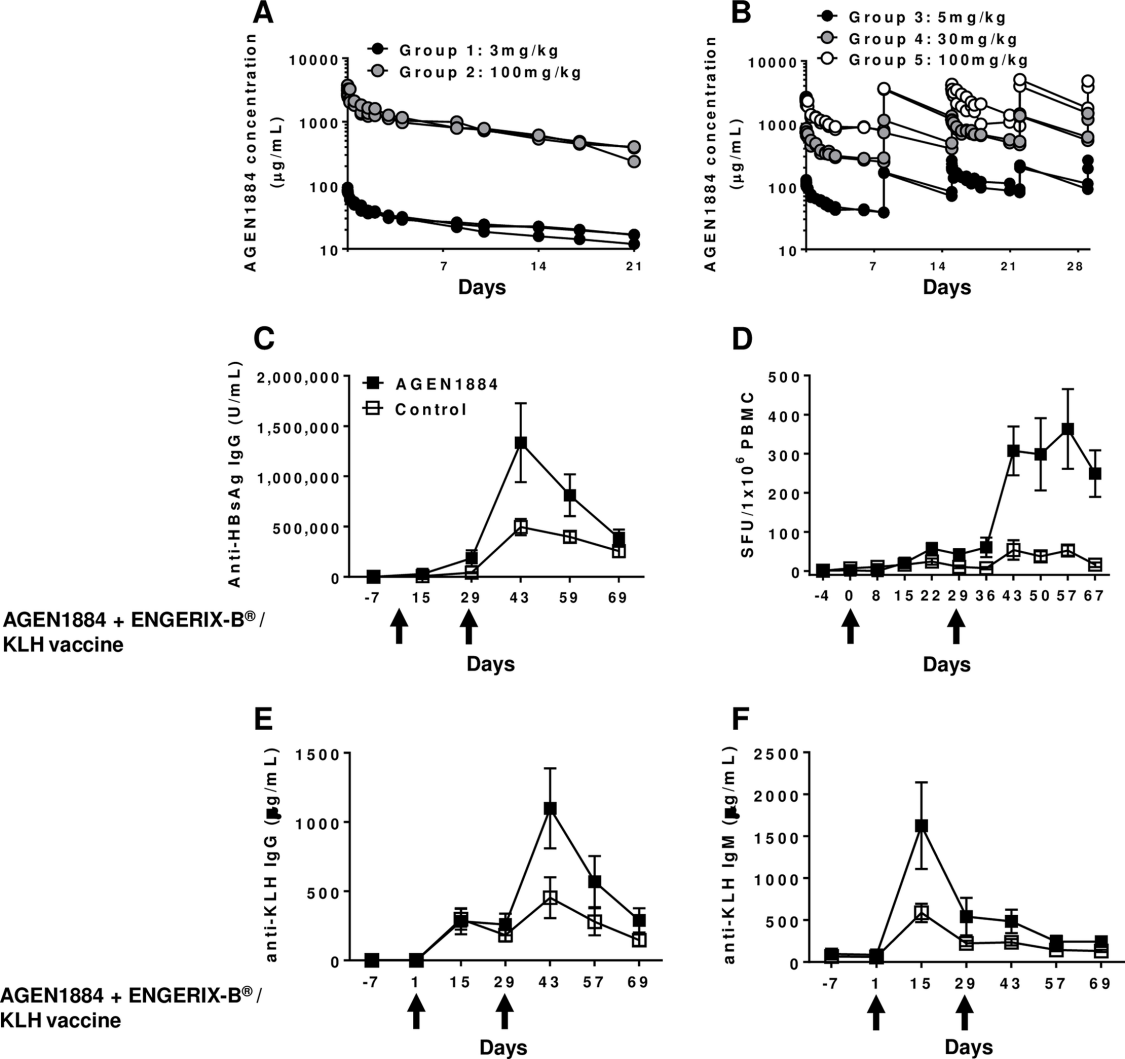
Pharmacokinetic profile of AGEN1884 and T cell-dependent antibody response and immune cell response to systemic co-administration of AGEN1884 with an ENGERIX-B® and KLH vaccine in cynomolgus macaques. AGEN1884 titers were measured from serum samples collected from five groups of animals (n = 3 per group) following (A) a single intravenous (IV) dose at either 3 mg/kg or 100 mg/kg or (B) five weekly IV doses at 5 mg/kg, 30 mg/kg, or 100 mg/kg. Cynomolgus macaques (n = 6 per group) were given 10 mg/kg of AGEN1884 *via* IV administration with an ENGERIX-B® and KLH vaccine on days 1 and 29. Duplicate samples were analyzed for (C) anti-HBsAg-specific IgG serum titers or (D) the frequency of IFN-γ-producing HBsAg-specific cells in PBMC based on the number of spot forming units (SFU) per million PBMC. Anti-KLH (E) IgG and (F) IgM serum titers were also measured.

AGEN1884 binds to recombinant cynomolgus macaque CTLA-4 (S1 Table). To evaluate the tolerability and PK profile of AGEN1884 in cynomolgus macaques, single (3 or 100 mg/kg) or multiple (5, 30 or 100 mg/kg) intravenous doses were administered (Fig 5A and 5B). All doses were generally well-tolerated, and plasma analyses showed dose-proportionate increases in systemic antibody exposure and a mean half-life of 8 to 14 days. There were no findings from clinical observations or physical, neurologic, ophthalmic or electrocardiographic examinations that were considered to be AGEN1884-related. These data demonstrate that systemic administration of AGEN1884 was safe and well-tolerated in cynomolgus macaques up to 100 mg/kg (the no-observed-adverse-effect-level).

Cytokine release assays (CRAs) are routinely used to evaluate the potential for cytokine release syndrome in patients infused with monoclonal antibodies [52]. AGEN1884 was added to human peripheral blood in either a plate-bound (i.e. complexed to mimic cross-linking) or soluble format. The median concentration for all cytokines evaluated (IL-2, IL-4, IL-6, IL-8, IL-10, IFN-γ and TNF-α) was below the limit of detection except for IL-6 and IL-8 at the highest concentration of soluble AGEN1884 (100 μg/mL). In a plate-bound (complexed) format, the median cytokine concentration elicited by AGEN1884 was zero (S6 Fig). Cetuximab, an anti-epidermal growth factor receptor (EGFR) monoclonal antibody, was included as a negative control and has been associated with a low incidence of infusion reactions. In contrast, two positive controls in this assay, SEB peptide and alemtuzumab (anti-CD52 antibody), an antibody associated with cytokine release syndrome in some patients, yielded markedly high cytokine concentrations [35]. These findings suggest that AGEN1884 does not induce levels of cytokines in a human whole blood assay that would be predictive of cytokine release syndrome *in vivo* [53].

### Immune responses in cynomolgus macaques to reporter vaccines co-administered with AGEN1884

Enhancement of vaccine responses in non-human primates can be used to demonstrate the activity or potency of immunomodulatory antibodies. To evaluate the potential for AGEN1884 to augment cellular and humoral vaccine immunity, cynomolgus macaques were administered AGEN1884 at 10 mg/kg concurrently with the HBsAg vaccine (ENGERIX-B^®^) and KLH protein, a well-known immunogen [54]. AGEN1884 enhanced the anti-HBsAg IgG response (a T cell-dependent antibody response (TDAR)) compared to the vehicle control-treated animals, as well as the frequency of HBsAg-specific IFN-γ-producing immune cells (Fig 5C and 5D). Similarly, KLH-specific IgG and IgM serum titers were also increased on day 43 or 15, respectively, following AGEN1884 administration as compared to the vehicle control (Fig 5E and 5F). No differences in the levels of systemic cytokines, chemokines and pro-inflammatory factors were observed in animals dosed with AGEN1884 or the vehicle together with the HBsAg vaccine and KLH (S7 Fig). These data demonstrate that AGEN1884 enhances antigen-specific immune responses in combination with reporter vaccines *in vivo*, with no evidence of systemic cytokine release post-treatment.

### AGEN1884 cooperates with PD-1 blockade to enhance T cell proliferation in a non-human primate model

**Fig 6:**
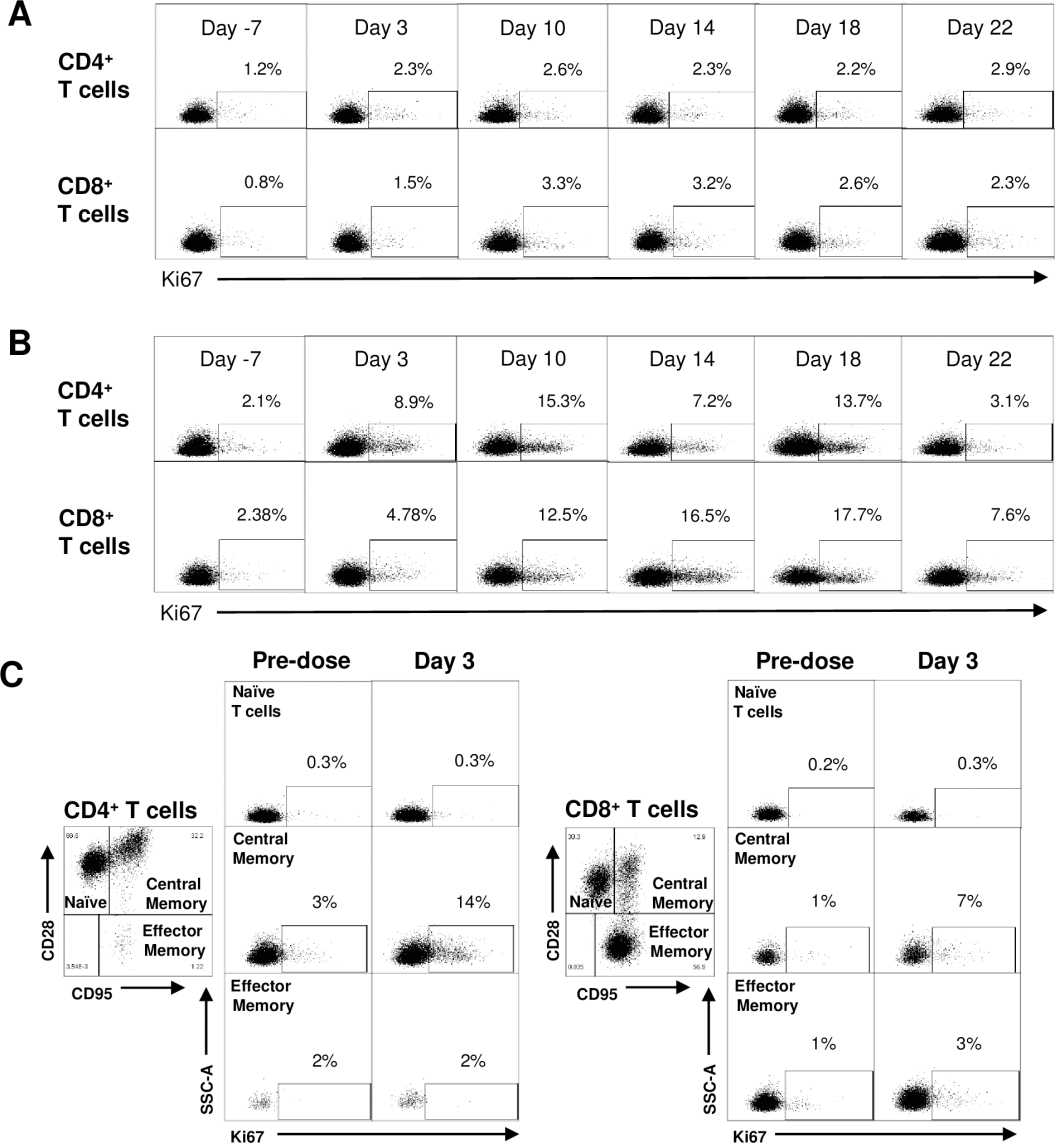
AGEN1884 in combination with anti-PD-1 further potentiates T cell proliferation *in vivo*. Cynomolgus macaques (n = 6 per group) were administered 3 mg/kg of nivolumab alone or in combination with 10 mg/kg of AGEN1884 *via* intravenous (IV) infusion. Duplicate samples of PBMC were analyzed for Ki67 expression in CD4^+^ and CD8^+^ T cells at 7 days prior to treatment or 3, 10, 14, 18 and 22 days after treatment. (A-B) Representative dot plots of CD4^+^ and CD8^+^ T cells isolated from PBMC from cynomolgus macaques given (A) 3 mg/kg of nivolumab alone or (B) a combination of 10 mg/kg of AGEN1884 with 3 mg/kg of nivolumab. (C) Representative dot plots of naïve, central memory and effector memory CD4^+^ and CD8^+^ T cells isolated from PBMC from cynomolgus macaques given a combination of 10 mg/kg of AGEN1884 with 3 mg/kg of nivolumab.

Clinical trials of anti-CTLA-4 (ipilimumab) and PD-1 (nivolumab) antibodies in combination have shown evidence of a PD effect on peripheral T cell proliferation [44, 45]. Similarly, a study combining anti-PD-L1 (durvalumab) and CTLA-4 (tremelimumab) antibodies produced a similar PD response [55]. The proliferation marker, Ki67, is routinely measured in flow cytometry assays, and is present only in the active phases of the cell cycle [56]. To determine if AGEN1884 can promote a T cell-associated proliferative PD response in combination with an anti-PD-1 antibody, cynomolgus macaques were administered a single dose (10 mg/kg) of AGEN1884 together with the anti-PD-1 antibody, nivolumab (3 mg/kg) (Fig 6). Simultaneous blockade of the CTLA-4 and PD-1 pathways by AGEN1884 and nivolumab showed qualitative evidence of enhanced CD4^+^ and CD8^+^ central memory T (T_CM_) cell proliferation in some animals as compared to the anti-PD-1 monotherapy group (Fig 6A–6C). Similar to anti-PD-1 monotherapy, AGEN1884 alone did not produce a measurable increase in Ki67 expression in CD4^+^ or CD8^+^ T cells in a separate study (S8 Fig). Together, our findings suggest that AGEN1884 is pharmacologically active in non-human primates, and combines with PD-1 pathway blockade to elicit a dynamic T cell-associated proliferative PD response. Based on previous studies, this PD assessment may be translated into patients to explore various dose regimens, particularly AGEN1884 in combination with anti-PD-1 or PD-L1 antibodies.

### AGEN2041, the IgG2 variant of AGEN1884, increased vaccine-specific antibody and T cell responses

**Fig 7:**
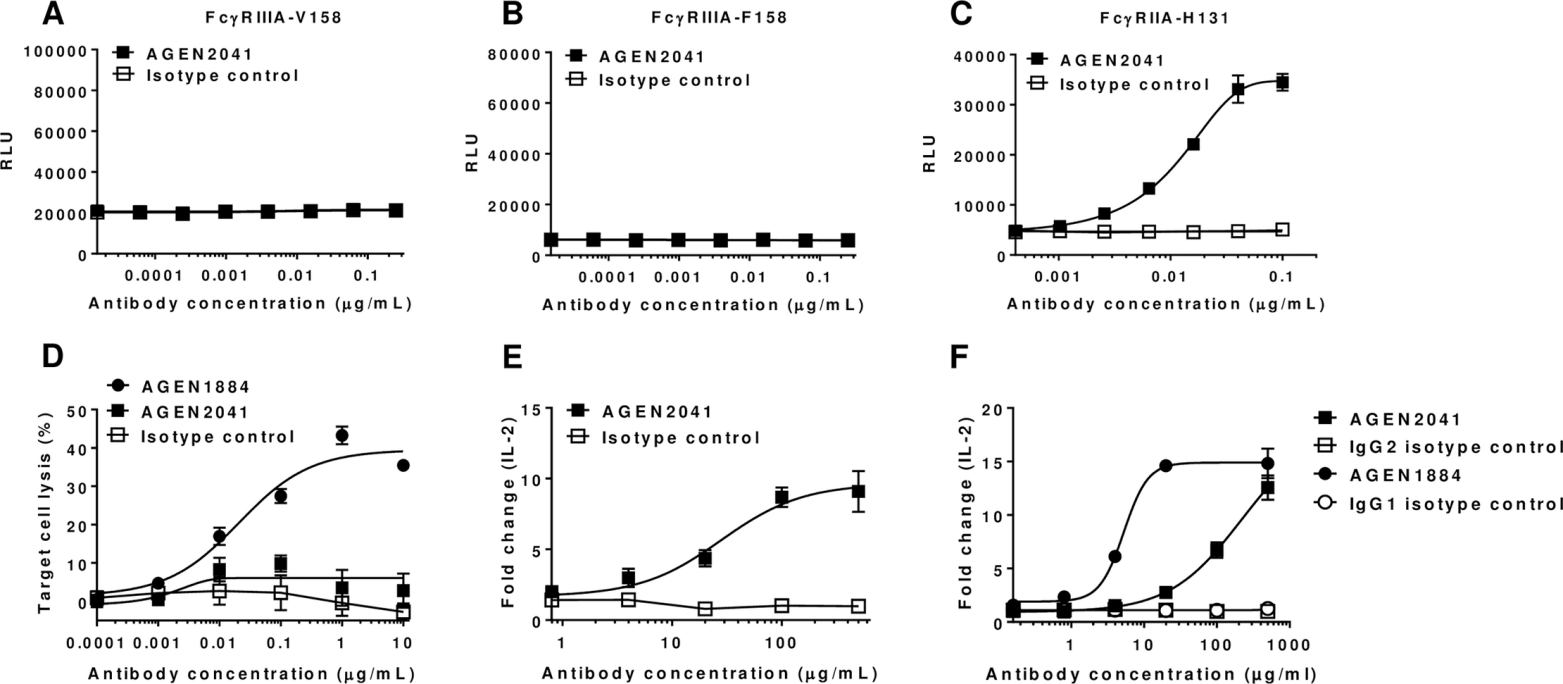
Fcγ receptor signaling and antibody-dependent cellular cytotoxicity of CTLA-4-expressing cells with AGEN2041 and increased T cell activation in the presence of AGEN2041. (A-C) Jurkat cell lines genetically engineered to express FcγRs upstream of a NFAT-dependent luciferase reporter were co-cultured with CTLA-4-expressing cells and increasing doses of AGEN2041 or an isotype control. Signaling through (A) FcγRIIIA-V158-, (B) FcγRIIIA-F158-, or (C) FcγRIIA-H131-expressing cells was assessed based upon luciferase expression which is shown as relative light units (RLU). (D) The cytotoxicity of CTLA-4^high^ Jurkat cells by CD16-expressing NK-92 cells based on lactate dehydrogenase (LDH) release in the presence of increasing concentrations of AGEN2041, AGEN1884 (positive control), or an IgG2 isotype control antibody. (E-F) Primary human PBMC were stimulated with a sub-maximal concentration of the SEA peptide (100 ng/mL) and increasing doses of (E) AGEN2041 versus (F) AGEN1884. Cell supernatants were collected after 5 days for measurement of IL-2. Representative data indicate the mean ± SEM in each treatment group (n = ≥2).

In addition to ipilimumab, an IgG2 anti-CTLA-4 antibody, tremelimumab, is currently in clinical trials in different combination regimens for solid tumor malignancies [55]. Whether differences in the Fc region might impart unique functional attributes remains to be established. Therefore, we sought to compare AGEN1884 with its IgG2 Fc variant, AGEN2041. Importantly, AGEN2041 and AGEN1884 share identical heavy chain and light chain variable regions, bound to human CTLA-4 with similar affinity and selectivity and showed equivalent blockade of CD80 and CD86 binding to CTLA-4, with a half-maximal effective concentration (EC50) of 0.05 μg/mL compared to AGEN1884 at 0.03 μg/mL (S9A–S9C Fig). Consistent with an IgG2 Fc region, AGEN2041 showed reduced binding to activating FcγRs, but could retain binding to the high affinity variant of FcγRIIA (H131). This observation is consistent with the possibility that IgG2 isotype antibodies may retain some potential to elicit ADCP against antibody-bound target cells (S5A–S5F Fig). Consistent with this binding profile, AGEN2041-labeled target cells did not mediate significant FcγRIIIA signaling or NK cell-mediated ADCC. However, AGEN2041 could signal through FcγRIIA when bound to antigen-expressing target cells (Fig 7A–7D). Commensurate with the retained features of the variable binding domain, AGEN2041 enhanced IL-2 production in the SEA peptide assay (Fig 7E). Surprisingly, when compared directly with AGEN1884 in the same APC co-culture assay, we observed approximately a 40-fold difference in the EC50 (Fig 7F). This increase in potency of AGEN1884 compared to AGEN2041 was observed across multiple donors (S9D–S9F Fig). Notably, higher concentrations (above 100 μg/mL) of AGEN2041 achieved a similar level of T cell-produced IL-2 to that of AGEN1884.

Finally, we assessed the immunomodulatory potential AGEN2041 in the same reporter vaccine non-human primate model. Similar to AGEN1884, AGEN2041 had a mean half-life of 8 to 13 days and was well tolerated (up to 100 mg/kg), with no evidence of systemic cytokine release in the peripheral blood (S10 Fig). At 10 mg/kg, AGEN1884 and AGEN2041 both combined effectively with ENGERIX-B^®^ to produce a similar increase in the anti-HBsAg IgG response (Fig 5 and S11 Fig). Therefore, at equivalent doses of 10 mg/kg, AGEN1884 and AGEN2041 both enhanced vaccine-specific immune responses. It remains unclear if at lower doses the profound differences between the IgG1 and IgG2 variants *in vitro* might translate *in vivo*. Further, a mechanistic explanation that could attest for the differences in IgG1 versus IgG2 molecules remains to be elucidated, in particular as they relate to FcγR biology.

## Discussion

The CTLA-4 and PD-1 pathways function cooperatively to limit T cell activation during priming by APCs leading to reduced proliferation, cytokine and chemokine production and cell survival [57]. CTLA-4 is rapidly translocated from intracellular vesicles to the cell surface following TCR triggering, where it can potently attenuate T cell differentiation, primarily by preventing CD80 and CD86 on APCs from engaging the co-stimulatory receptor, CD28 [57]. Compared to CD28, CTLA-4 has a relatively higher affinity for CD80 and CD86, which enables it to effectively compete for these shared ligands [8, 9]. In the context of tumor-specific T cell immune responses, CTLA-4 is understood to negatively regulate T cell priming events in secondary lymphoid tissues, leading to an unresponsive, anergic or apoptotic phenotype [57, 58]. By contrast, PD-1 is upregulated on recently activated T cells after CTLA-4 has already participated in conditioning early events within the immune synapse [57]. Importantly, high levels of PD-1 surface expression are maintained on populations of chronically stimulated (“exhausted”) T cells, which has been documented for tumor-specific T cells that reside within the tumor microenvironment [57]. Unlike CD80 and CD86, the PD-1 ligands (PD-L1 and PD-L2) are not only expressed by APCs, but also by other immune cell types as well as tumor cells [59]. These observations support the hypothesis that kinetically, CTLA-4 acts as an early regulator of T cell function, while PD-1 may act more broadly to impair T cell activation, differentiation and survival as well as contribute to sustained T cell dysfunction within the tumor [59]. Consistent with this, AGEN1884 potently modulated T cell priming under conditions of suboptimal T cell activation. However, it also showed compelling combination activity *in vitro* with several anti-PD-1 antibodies, including nivolumab, pembrolizumab and a novel anti-PD-1 antagonist antibody, AGEN2034.

The interconnection between the CTLA-4 and PD-1 pathways is reinforced by the recent observation that PD-1 can directly dephosphorylate CD28 *via* the recruitment of the Src homology 2 domain-containing protein tyrosine phosphatase (SHP-2) [60]. This finding further supports the notion that simultaneous blockade of the CTLA-4 and PD-1 pathways may be required to mitigate T cell suppression and invoke effective anti-tumor immune responses. This perspective aligns with the clinical success of concomitant administration of anti-PD-1 and CTLA-4 antibodies in melanoma and lung cancer patients versus monotherapy [20, 61]. Notably, the co-administration of PD-1- and CTLA-4-targeted antibodies has also been correlated with gene expression and proliferation analyses of peripheral T cells, which were not seen with sequential dosing regimens [45]. The correlation between these PD effects on peripheral T cells and anti-tumor efficacy remains to be established. Here we could reproduce a peripheral T cell PD effect in animals that received AGEN1884 concurrently with an anti-PD-1 antibody. Notably, this T cell proliferative response was restricted to the central memory compartment, which is consistent with the selective activation of antigen-experienced versus naive T cells. In cancer patients, this population would include tumor-reactive T cell clones, including those against immunogenic tumor-associated neoepitopes generated by non-synonymous nucleotide mutations [62]. Further interrogation of TCR repertoire changes in the expanding populations of T cells, and whether this is restricted to a clonal versus polyclonal T_CM_ cell response, as well as how anti-PD-1 and CTLA-4 antibody exposure levels might impact the proliferation frequency and TCR clonality remains to be explored. On a related note, it has been previously shown that CTLA-4 blockade in melanoma patients can broaden the repertoire of tumor-specific CD8^+^ T cells compared to the pre-existing viral-specific T cell repertoire detected at the start of therapy [63]. How modulation of the PD-1 pathway together with CTLA-4 blockade alters tumor-specific versus other memory T cell responses remains to be examined.

CTLA-4 contributes to the immune suppressive function of Treg cells. One such mechanism involves the removal of CD80 and CD86 from the surface of APCs following CTLA-4 co-engagement on Treg cells [11]. Therefore, in addition to the ability of AGEN1884 to modulate Teff cell activation, this antibody may also limit the impact of suppressive Treg cells within the tumor microenvironment. Related to Treg cells, CTLA-4 has been shown to be overexpressed on intratumoral populations of activated Treg cells, both in mouse preclinical models and in primary human tumors [15, 64]. Consistent with this finding, several recent studies have provided novel mechanistic insights into the mode of action of immunomodulatory antibodies and have defined a key role for the interaction of antibodies with FcγRs [15, 65, 66]. Preclinical studies in mice have shown that optimal anti-tumor activity of anti-CTLA-4 antagonist antibodies required co-engagement of FcγRs expressed by tumor-associated effector cells to selectively deplete CTLA-4-expressing intratumoral Treg cells [15–17, 47]. In accordance with this preclinical finding, a recent study in melanoma patients who had responded to a human IgG1 anti-CTLA-4 antibody (ipilimumab) found a correlation between increased FcγRIIIA-expressing non-classical monocytes in the peripheral blood and tumors and decreased intratumoral Treg cell infiltrates [22]. Based on our characterization of AGEN1884, the FcγR binding and functional properties of AGEN1884 may be ideally suited to take advantage of this mode of therapeutic activity in cancer patients.

AGEN2041 has identical variable regions to AGEN1884, but was designed with an IgG2 Fc region. As such, this antibody showed no ADCC potential in FcγRIIIA binding and signaling assays but did retain an interaction with FcγRIIA, which may enable ADCP functionality [67]. To our knowledge, this is the first head-to-head functional comparison of an IgG1 and IgG2 anti-CTLA-4 antibody. Administered at 10 mg/kg, AGEN2041 and AGEN1884 were both able to enhance vaccine-specific antibody responses in non-human primates. However, in a primary T cell assay, we observed a striking 40-fold difference in potency between AGEN1884 and AGEN2041. It has been previously demonstrated that human antibodies have different affinities for human and non-human primate FcγRs, including the higher affinity human IgG2 has for macaque versus human FcγRIIB [68, 69]. Whether increased human IgG2 binding to macaque FcγRIIB, which contains the high affinity H131 residue, affects the potency of AGEN2041 in non-human primates compared to the human *in vitro* assays remains to be investigated. Moreover, how these differences may translate into the clinical setting remains unclear, including whether a titration of AGEN1884 versus AGEN2041 *in vivo* could resolve the differences in pharmacological activity observed in immune cell *in vitro* assays. Interestingly, ipilimumab is reported to have a half-life of 12–14 days, as compared to 22 days for tremelimumab. Despite its longer half-life, PK simulations for tremelimumab predicted that a 15 mg/kg dose every 3 months achieved the intended target concentration in only 50% of patients [70]. This compares with 90% in patients dosed at 10 mg/kg every 4 weeks for ipilimumab. Notably, both tremelimumab and ipilimumab continue to be investigated at different doses and in a variety of malignancies and combination regimens. These findings, together with our observations, suggest that IgG2 anti-CTLA-4 antibodies may need to be dosed at higher concentrations to achieve equivalent pharmacological modulation of the CTLA-4 pathway as compared to IgG1 anti-CTLA-4 antibodies.

Taken together, targeting the cell intrinsic and extrinsic mechanisms of the CTLA-4 pathway with neutralizing antibodies can be considered as a requisite approach to enable other therapeutic strategies aimed to promote or restore tumor-specific T cell immune responses in patients. This finding appears to be a unique feature of the CTLA-4 pathway, with other co-inhibitory and co-stimulatory T cell pathways, including LAG-3 and T-cell immunoglobulin domain and mucin domain 3 (TIM-3), playing a secondary role to further sculpt the magnitude and durability of the T cell response and potentially address resistance mechanisms within the tumor microenvironment [71]. Herein, we have characterized AGEN1884, a novel human IgG1 anti-CTLA-4 antagonist antibody that has been engineered to harness several potential *in vivo* mechanisms of action including: i) interference with CTLA-4 binding to CD80 and CD86, allowing for T cell co-stimulation *via* CD28; ii) blockade of CD80 and CD86 trans-endocytosis by CTLA-4-expressing Treg cells; iii) selective depletion of CTLA-4-expressing intratumoral Treg cells, by virtue of its human IgG1 Fc region; and iv) the versatility to combine effectively with other antibodies targeting co-inhibitory or co-stimulatory pathways to further improve the magnitude and durability of tumor-specific T cell immune responses to cancer-associated antigens [18, 62]. Collectively, the pharmacological properties of AGEN1884 warrant in-human testing. AGEN1884 is currently in early clinical evaluation as a single agent, and is intended to be evaluated in combination with anti-PD-1 antagonist antibodies, including AGEN2034.

## Supporting information

S1 Table AGEN1884 binding affinity to human, cynomolgus macaque and rodent CTLA-4. To evaluate the cross-species binding, AGEN1884 was captured on a sensor chip using an anti-human Fab antibody reagent, and binding to recombinant human or cynomolgus macaque CTLA-4 Fc fusion proteins was assessed in solution phase by surface plasmon resonance (SPR). Average estimated k_a_ (association rate constant), k_d_ (dissociation rate constant) and equilibrium dissociation constant (K_D_) calculated by SPR for AGEN1884 binding to recombinant His-tagged human CTLA-4 or Fc-tagged human, cynomolgus macaques or mouse CTLA-4. LOD: level of detection.
(TIF)

S1 Fig AGEN1884 selected from 20 different clones to block CD80 and CD86 binding to CTLA-4. Recombinant human CTLA-4-Fc was coupled to microsphere beads and incubated with a titrated dose of 20 different clones, including AGEN1884 (thick black line) for one hour at room temperature. Fluorescently labeled (A) CD80-Fc or (B) CD86-Fc fusion proteins (at 1 nM) were then added, and the percent of fluorescently-labeled CD80-Fc or CD86-Fc binding to the microspheres was determined.
(TIF)

S2 Fig AGEN1884 selectively binds to human and cynomolgus macaque CTLA-4, but not related CD28 family members. (A-B) Microsphere beads were coupled to the indicated CD28 family member and incubated with AGEN1884 (8.3 mg/mL). (A) AGEN1884 or (B) an isotype control IgG1 binding was detected using a fluorochrome-conjugated anti-human IgG secondary antibody. The mean fluorescence intensity (MFI) was determined based upon the unique spectral signature of the microspheres and quantified using a fluorescent plate reader. Representative data from at least two independent experiments are shown above. (C-D) SPR affinity measurement of AGEN1884, which was immobilized on a CM5 sensor chip, and either (C) CTLA-4-Fc or (D) CD28-Fc were independently run over the chip at increasing concentrations using a Biacore T200.
(TIF)

S3 Fig AGEN1884 engagement of CTLA-4 expressed by activated human T cells does not impact T cell cytokine production. CD3-expressing T cells were isolated from human PBMC and stimulated with platebound anti-CD3 antibody (5 μg/mL) in the presence of increasing concentrations of either (A) soluble or (B) plate bound AGEN1884, and the percentage of CD8-expressing T cells secreting IFN-γ was determined using flow cytometry. As a control, cells were stimulated with increasing concentrations of an isotype control antibody (n = ≥2).
(TIF)

S4 Fig AGEN2034 blocks PD-L1 and PD-L2, binds PD-1 and increases T cell activation. Binding of fluorescently-labeled (A) PD-L1-Fc or (B) PD-L2-Fc (1 nM) in the presence of increasing concentrations of AGEN2034 or an IgG4 isotype control. Binding to PD-1-linked microspheres was assessed using Luminex. (C) AGEN2034 binding to PD-1^+^CD8^+^ T cells. (D) Primary human PBMC were stimulated with a sub-maximal concentration of the SEA peptide (100ng/mL) and increasing doses of AGEN2034. Cell supernatants were collected after 5 days for measurement of IL-2. Representative data indicate the mean ± SEM in each treatment group (n = ≥2).
(TIF)

S5 Fig The binding profiles of AGEN1884 to activating and inhibitory Fcγ receptors. (A-F) Binding of increasing doses of AGEN1884 or AGEN2041 (0.0005–30 μg/mL) to (A) rCHO-huFcγRIA-, (B) rJurkat-huFcγRIIA-H131-, (C) rCHO-huFcγRIIA-R131-, (D) rCHO-huFcγRIIIA-V158-, (E) rCHO-huFcγRIIIA-F158- and (F) rCHO-huFcγRIIB-expressing cell lines. The mean fluorescence intensity (MFI) was determined based on binding of an anti-F(ab’)2-PE labeled secondary F(ab’)2 fragment to AGEN1884 (black squares) compared to AGEN2041 (IgG2; white squares).
(TIF)

S6 Fig Median cytokine concentrations in response to AGEN1884. Fresh whole blood from ten donors was incubated with increasing concentrations (0.1, 1, 10, and 100 mg/mL) of (A) soluble or (B) plate-bound AGEN1884 in triplicate wells at 37°C and 5% CO_2_ for 24 hours. Plasma from each test set was isolated, pooled and replicates of 12 were tested for the presence of IL-2, IL-4, IL-6, IL-8, IL-10, IFN-γ and TNF-α. Data points represent the median concentration (pg/mL) in each treatment group. PBS or cetuximab were used as negative controls and alemtuzumab and Staphylococcal enterotoxin B (SEB) were used as positive controls for cytokine release. Median cytokine levels were zero except for (C) IL-6 and (D) IL-8 when 100 mg/mL of soluble AGEN1884 was tested.
(TIF)

S7 Fig Cytokine profile following AGEN1884 administration in cynomolgus macaques. Serum cytokines (A,B) IL-6, (C,D) IL-2, (E,F) IFN-γ and (G,H) IL-8, (I,J) IL-1β, (K,L) IL-7, (M,N) IL-10 and (O,P) TNF-β were measured pre-dose (0 hrs) and 2, 6 and 24 hrs post-infusion at (A,C,E,G,I,K,M,O) day 1 and (B,D,F,H,J,L,N,P) day 29 from two groups of cynomolgus macaques (n = 6 per group) treated with AGEN1884 or a control vehicle and vaccinated with KLH and HBsAg.
(TIF)

S8 Fig AGEN1884 alone does not potentiate T cell proliferation. Representative dot plots of CD4^+^ and CD8^+^ T cells isolated from PBMC from cynomolgus macaques treated intravenously with 10 mg/kg of AGEN1884. PBMC were analyzed for Ki67 expression in CD4^+^ and CD8^+^ T cells at four days prior to treatment or 15 and 22 days after treatment.
(TIF)

S9 Fig AGEN2041 binds CTLA-4 and blocks CTLA-4 from interacting with CD80 and CD86. (A-B) AGEN2041 binding to a (A) Jurkat cell line genetically engineered to express human CTLA-4 or (B) wildtype (CTLA-4-negative) Jurkat cell line. (C) Binding of fluorescently-labeled CD80-Fc or CD86-Fc (1 nM) in the presence of increasing concentrations of AGEN2041 or an IgG2 isotype control. Binding to CTLA-4-linked microspheres was assessed using Luminex. (D-F) Primary human PBMC were stimulated with a sub-maximal concentration of the SEA peptide (100 ng/mL) and a single dose of AGEN1884, AGEN2041 or an isotype control (10 μg/mL). Representative data indicate the mean ± SEM of multiple replicates in each treatment group (n = 3). Data were analyzed using a Student’s t-test. Significant differences depicted were p<0.01 (*).
(TIF)

S10 Fig Cytokine profile following AGEN2041 administration in cynomolgus macaques. Serum cytokines (A,B) IL-6, (C,D) IL-2, (E,F) IFN-γ and (G,H) IL-8, (I,J) IL-1β, (K,L) IL-7, (M,N) IL-10 and (O,P) TNF-β were measured pre-dose (0 hrs) and 2, 6 and 24 hrs post-infusion at (A,C,E,G,I,K,M,O) day 1 and (B,D,F,H,J,L,N,P) day 29 from two groups of cynomolgus macaques (n = 6 per group) vaccinated with 10 mg/kg of AGEN2041 or a control vehicle in addition to a KLH and HBsAg vaccine.
(TIF)

S11 Fig T cell-dependent antibody response to systemic co-administration of AGEN2041 with an ENGERIX-B^®^ and KLH vaccine. Cynomolgus macaques (n = 6 per group) were administered 10 mg/kg of AGEN2041 *via* intravenous (IV) administration with an ENGERIX-B^®^ and KLH vaccine on days 1 and 29. Duplicate samples were analyzed for anti-HBsAg-specific IgG serum titers in PBMC.
(TIF)
