# Supplementary figures and images for: Toxicological and pharmacological assessment of AGEN1884, a novel human IgG1 anti-CTLA-4 antibody
Source: PLoS One. 2018 Apr 4;13(4):e0191926. doi: 10.1371/journal.pone.0191926 (PMC5884502; doi:10.1371/journal.pone.0191926)

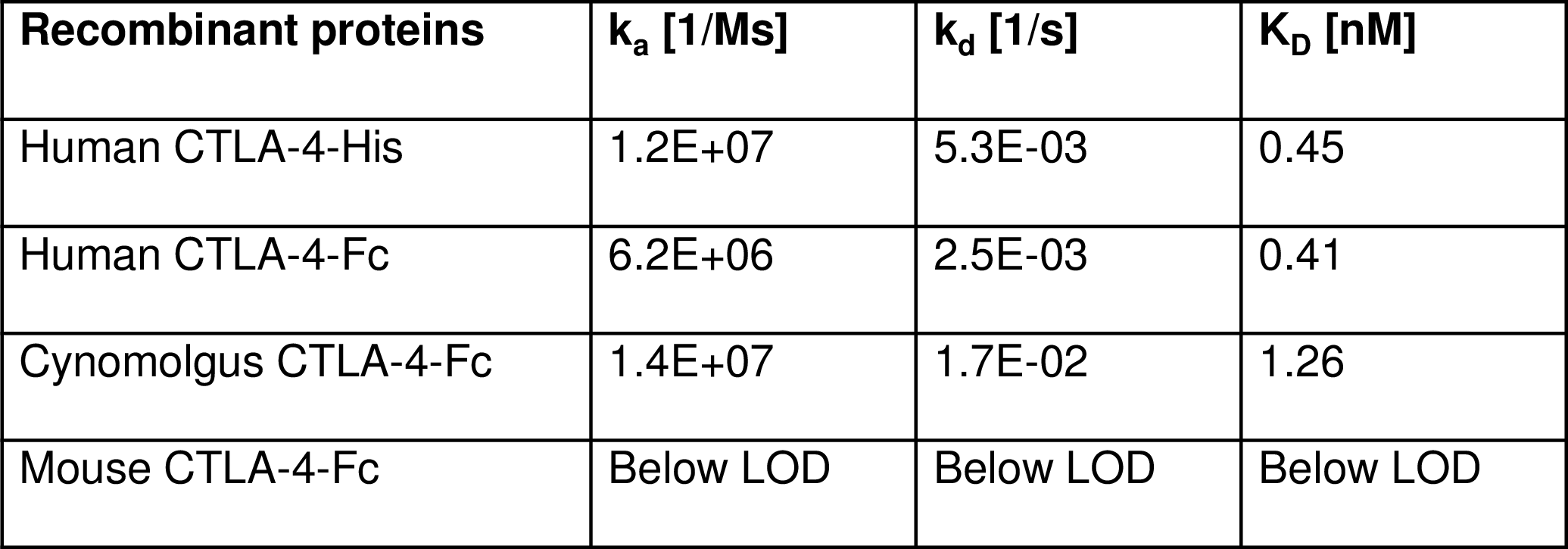

Supplement: S1 Table — To evaluate the cross-species binding, AGEN1884 was captured on a sensor chip using an anti-human Fab antibody reagent, and binding to recombinant human or cynomolgus macaque CTLA-4 Fc fusion proteins was assessed in solution phase by surface plasmon resonance (SPR). Average estimated ka (association rate constant), kd (dissociation rate constant) and equilibrium dissociation constant (KD) calculated by SPR for AGEN1884 binding to recombinant His-tagged human CTLA-4 or Fc-tagged human, cynomolgus macaques or mouse CTLA-4. LOD: level of detection. (TIF) [file pone.0191926.s001.tif]

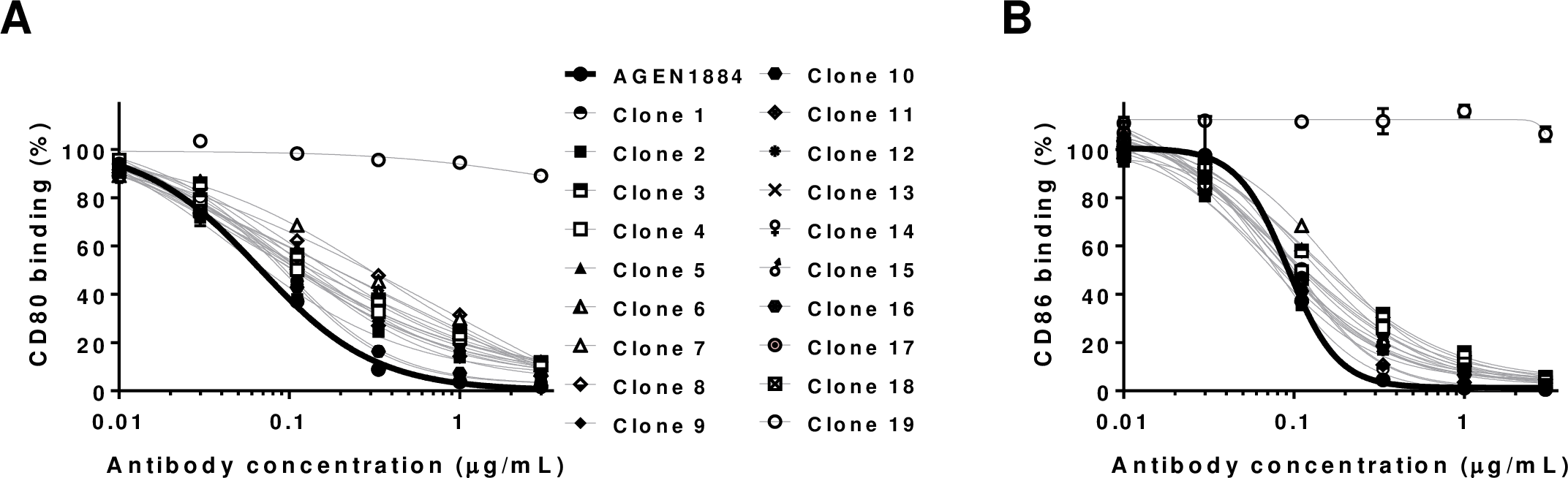

Supplement: S1 Fig — Recombinant human CTLA-4-Fc was coupled to microsphere beads and incubated with a titrated dose of 20 different clones, including AGEN1884 (thick black line) for one hour at room temperature. Fluorescently labeled (A) CD80-Fc or (B) CD86-Fc fusion proteins (at 1 nM) were then added, and the percent of fluorescently-labeled CD80-Fc or CD86-Fc binding to the microspheres was determined. (TIF) [file pone.0191926.s002.tif]

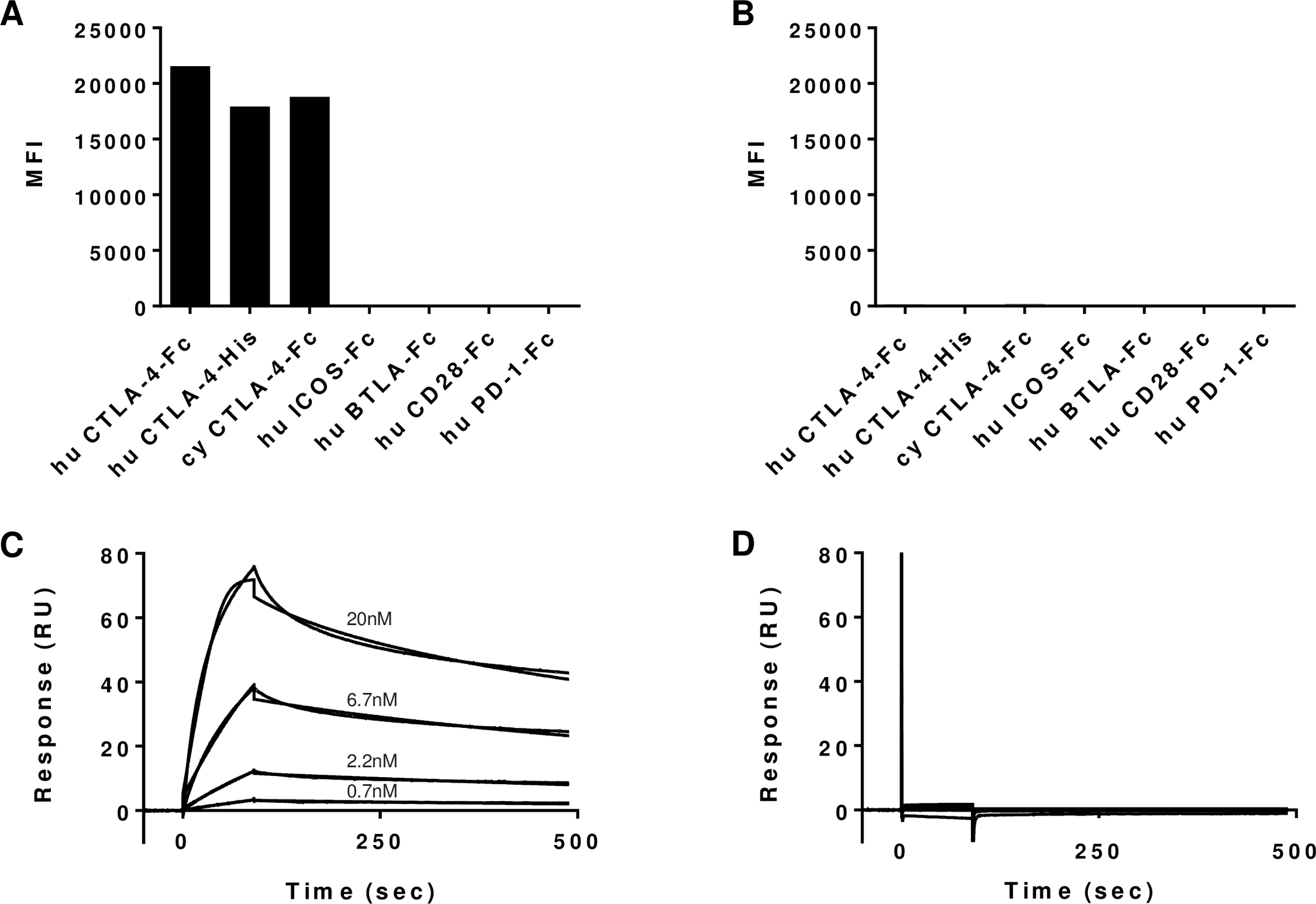

Supplement: S2 Fig — (A-B) Microsphere beads were coupled to the indicated CD28 family member and incubated with AGEN1884 (8.3 mg/mL). (A) AGEN1884 or (B) an isotype control IgG1 binding was detected using a fluorochrome-conjugated anti-human IgG secondary antibody. The mean fluorescence intensity (MFI) was determined based upon the unique spectral signature of the microspheres and quantified using a fluorescent plate reader. Representative data from at least two independent experiments are shown above. (C-D) SPR affinity measurement of AGEN1884, which was immobilized on a CM5 sensor chip, and either (C) CTLA-4-Fc or (D) CD28-Fc were independently run over the chip at increasing concentrations using a Biacore T200. (TIF) [file pone.0191926.s003.tif]

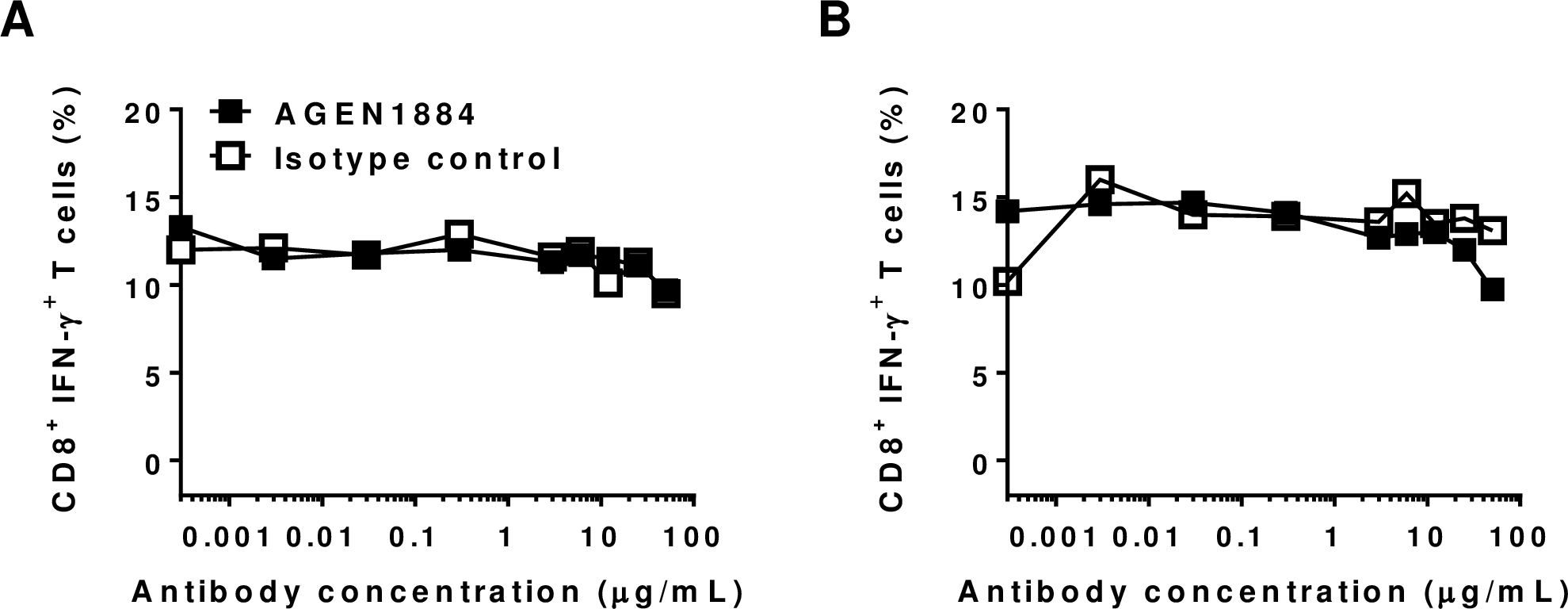

Supplement: S3 Fig — CD3-expressing T cells were isolated from human PBMC and stimulated with platebound anti-CD3 antibody (5 μg/mL) in the presence of increasing concentrations of either (A) soluble or (B) plate bound AGEN1884, and the percentage of CD8-expressing T cells secreting IFN-γ was determined using flow cytometry. As a control, cells were stimulated with increasing concentrations of an isotype control antibody (n = ≥2). (TIF) [file pone.0191926.s004.tif]

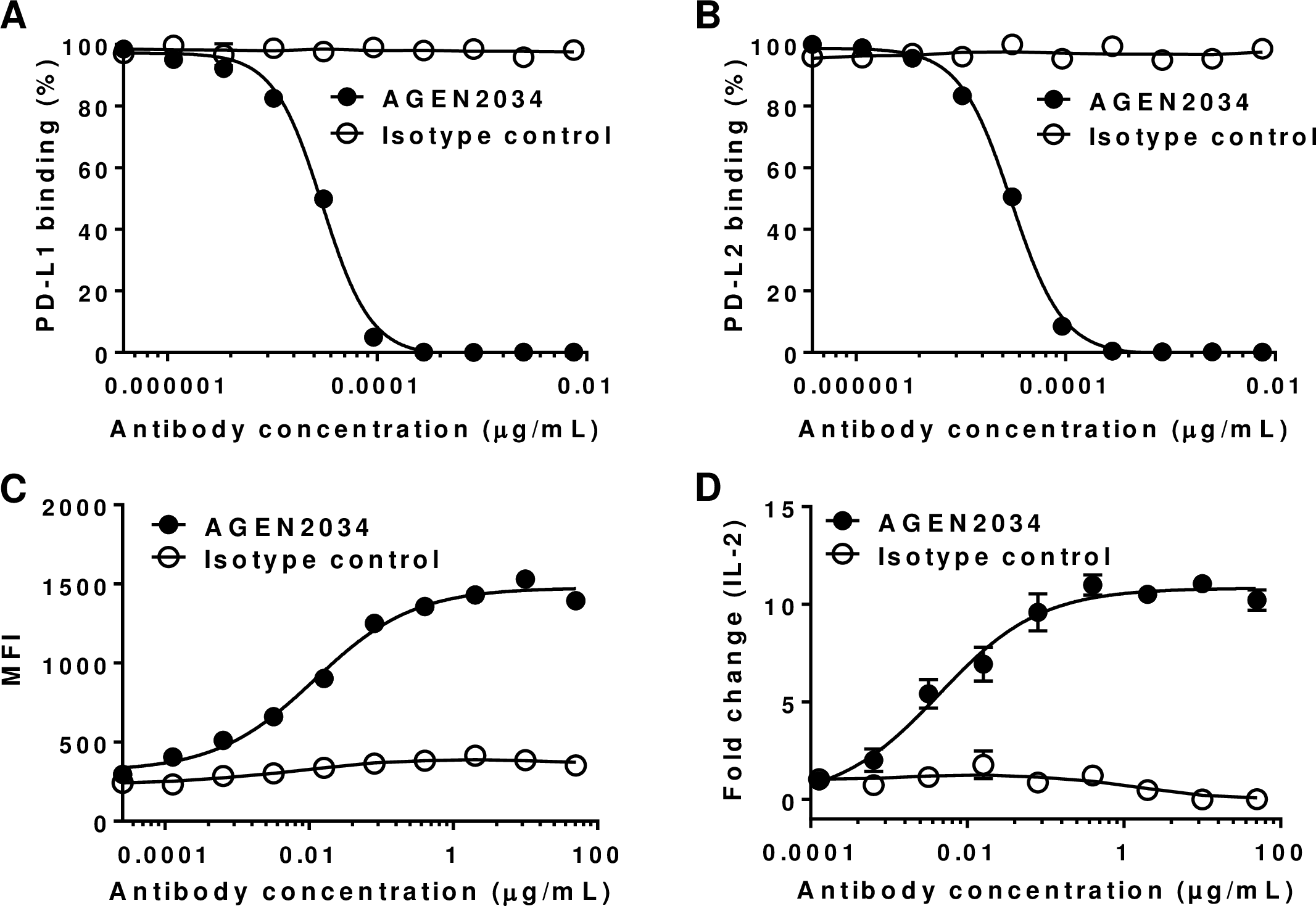

Supplement: S4 Fig — Binding of fluorescently-labeled (A) PD-L1-Fc or (B) PD-L2-Fc (1 nM) in the presence of increasing concentrations of AGEN2034 or an IgG4 isotype control. Binding to PD-1-linked microspheres was assessed using Luminex. (C) AGEN2034 binding to PD-1+CD8+ T cells. (D) Primary human PBMC were stimulated with a sub-maximal concentration of the SEA peptide (100ng/mL) and increasing doses of AGEN2034. Cell supernatants were collected after 5 days for measurement of IL-2. Representative data indicate the mean ± SEM in each treatment group (n = ≥2). (TIF) [file pone.0191926.s005.tif]

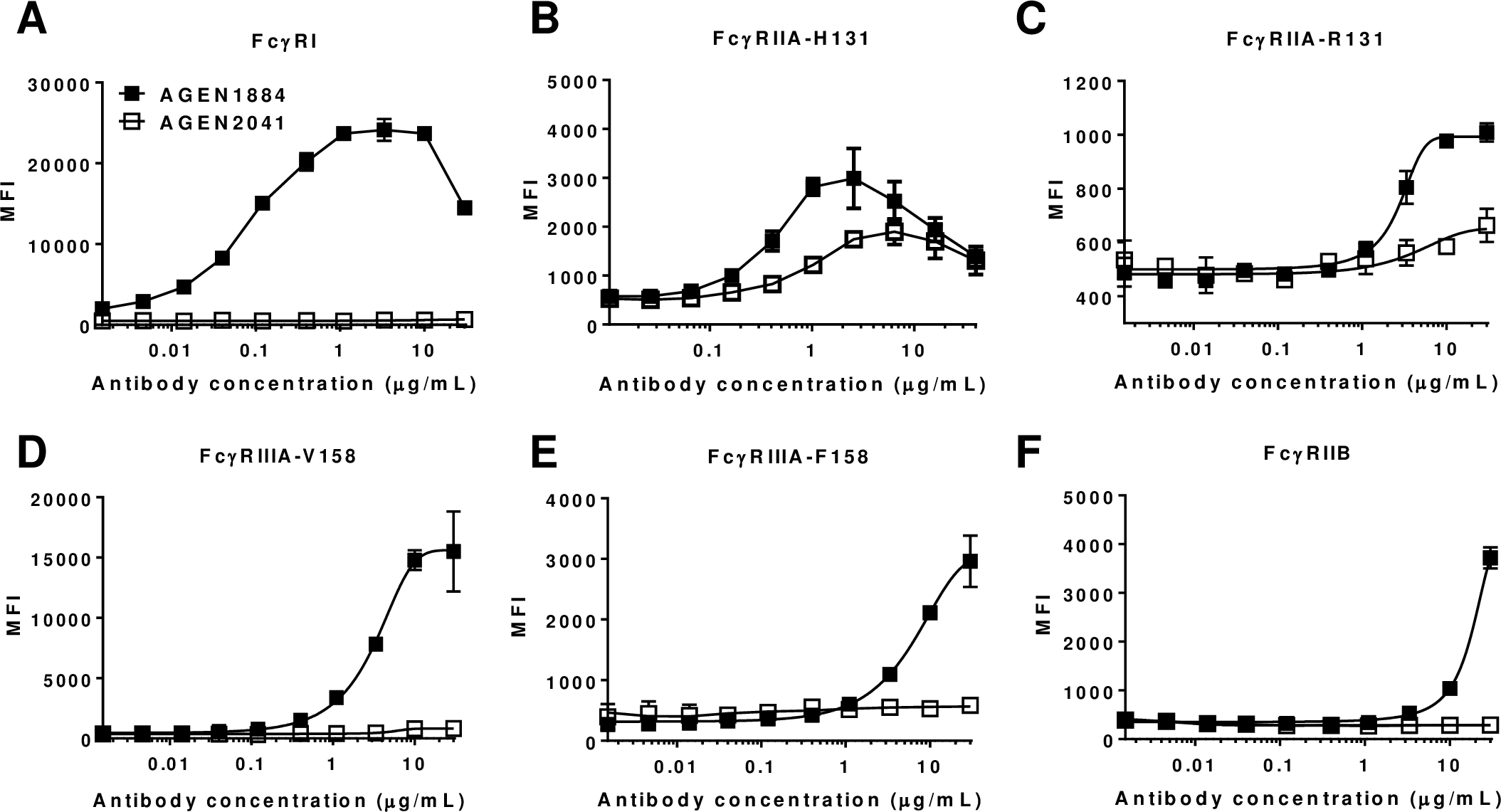

Supplement: S5 Fig — (A-F) Binding of increasing doses of AGEN1884 or AGEN2041 (0.0005–30 μg/mL) to (A) rCHO-huFcγRIA-, (B) rJurkat-huFcγRIIA-H131-, (C) rCHO-huFcγRIIA-R131-, (D) rCHO-huFcγRIIIA-V158-, (E) rCHO-huFcγRIIIA-F158- and (F) rCHO-huFcγRIIB-expressing cell lines. The mean fluorescence intensity (MFI) was determined based on binding of an anti-F(ab’)2-PE labeled secondary F(ab’)2 fragment to AGEN1884 (black squares) compared to AGEN2041 (IgG2; white squares). (TIF) [file pone.0191926.s006.tif]

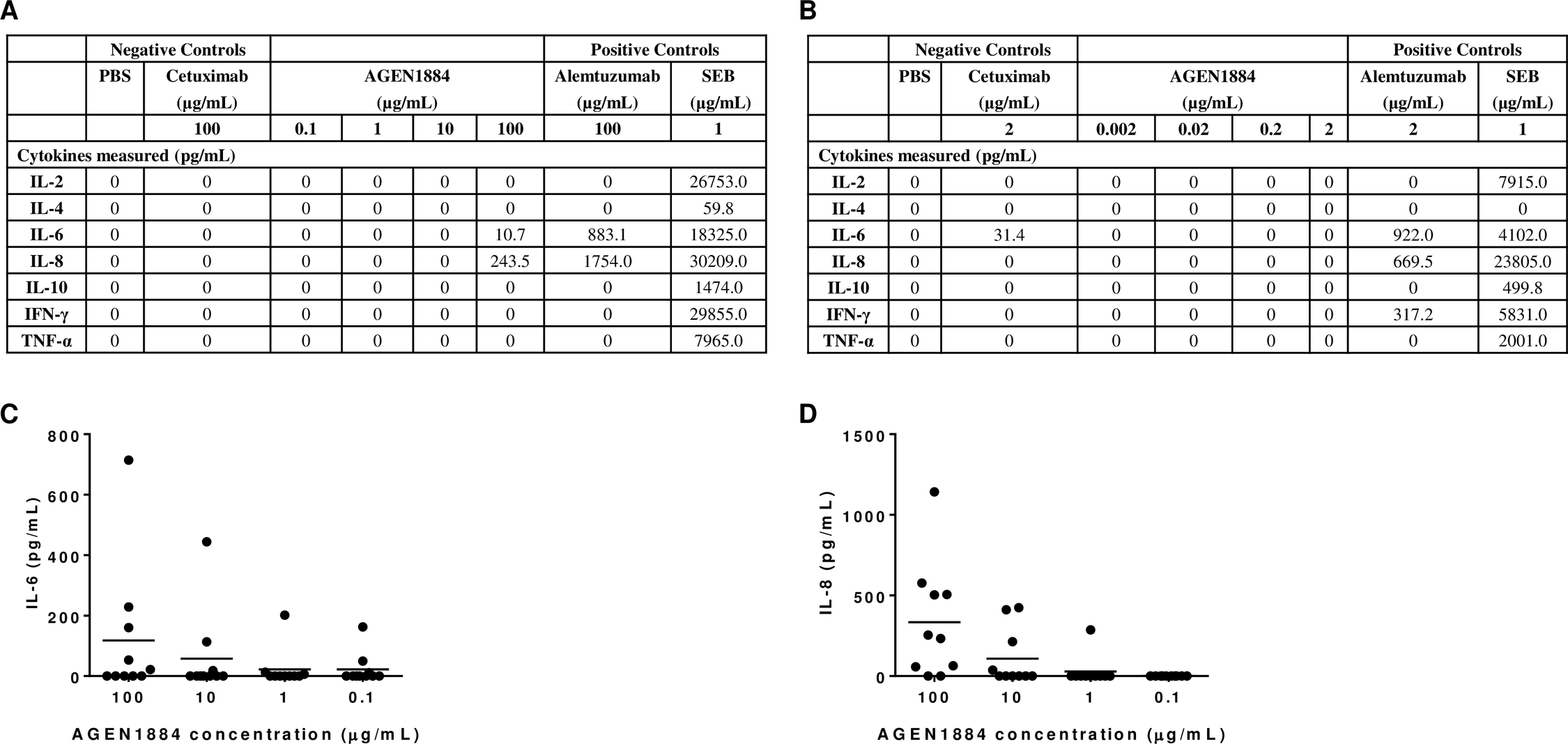

Supplement: S6 Fig — Fresh whole blood from ten donors was incubated with increasing concentrations (0.1, 1, 10, and 100 mg/mL) of (A) soluble or (B) plate-bound AGEN1884 in triplicate wells at 37°C and 5% CO2 for 24 hours. Plasma from each test set was isolated, pooled and replicates of 12 were tested for the presence of IL-2, IL-4, IL-6, IL-8, IL-10, IFN-γ and TNF-α. Data points represent the median concentration (pg/mL) in each treatment group. PBS or cetuximab were used as negative controls and alemtuzumab and Staphylococcal enterotoxin B (SEB) were used as positive controls for cytokine release. Median cytokine levels were zero except for (C) IL-6 and (D) IL-8 when 100 mg/mL of soluble AGEN1884 was tested. (TIF) [file pone.0191926.s007.tif]

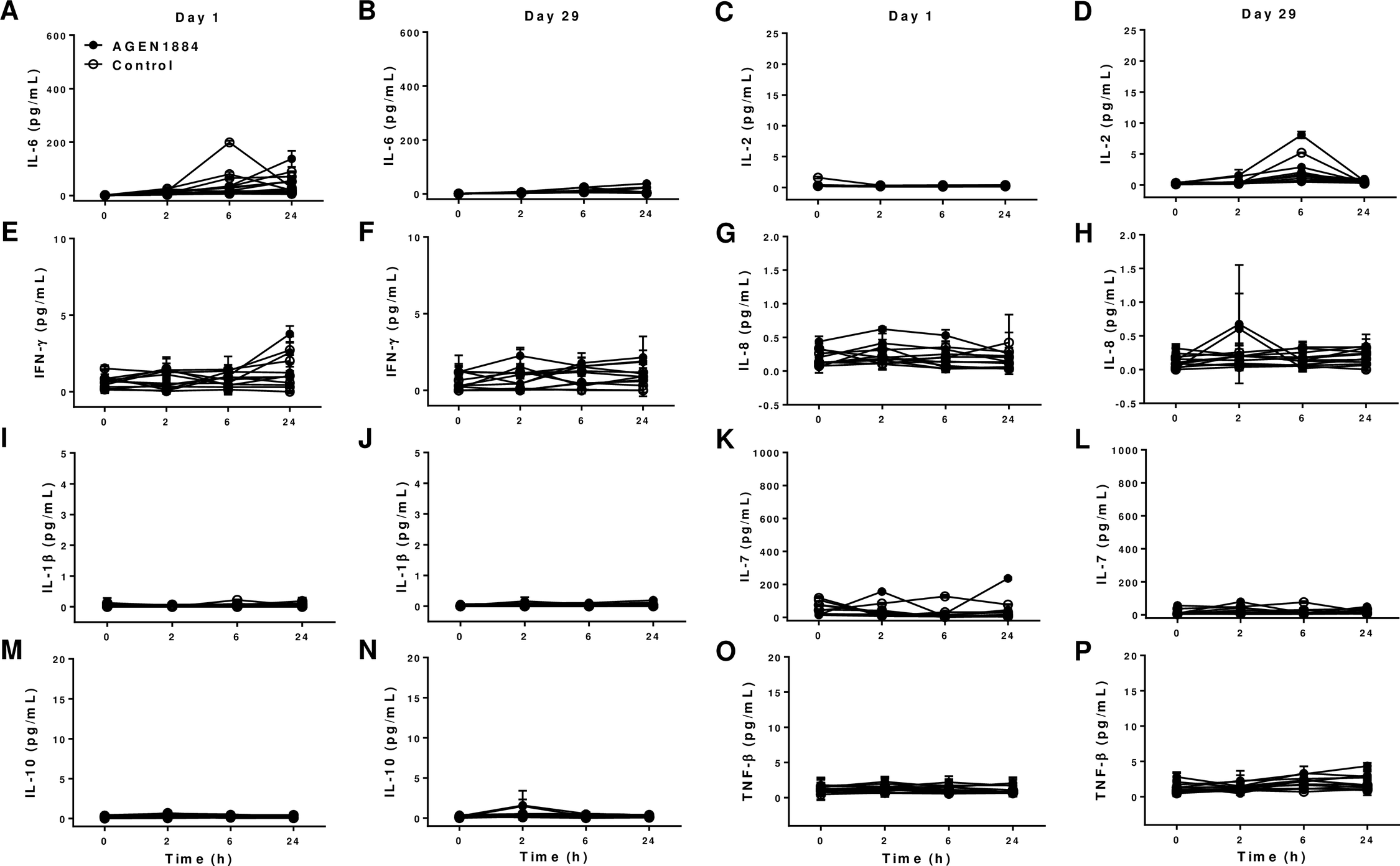

Supplement: S7 Fig — Serum cytokines (A,B) IL-6, (C,D) IL-2, (E,F) IFN-γ and (G,H) IL-8, (I,J) IL-1β, (K,L) IL-7, (M,N) IL-10 and (O,P) TNF-β were measured pre-dose (0 hrs) and 2, 6 and 24 hrs post-infusion at (A,C,E,G,I,K,M,O) day 1 and (B,D,F,H,J,L,N,P) day 29 from two groups of cynomolgus macaques (n = 6 per group) treated with AGEN1884 or a control vehicle and vaccinated with KLH and HBsAg. (TIF) [file pone.0191926.s008.tif]

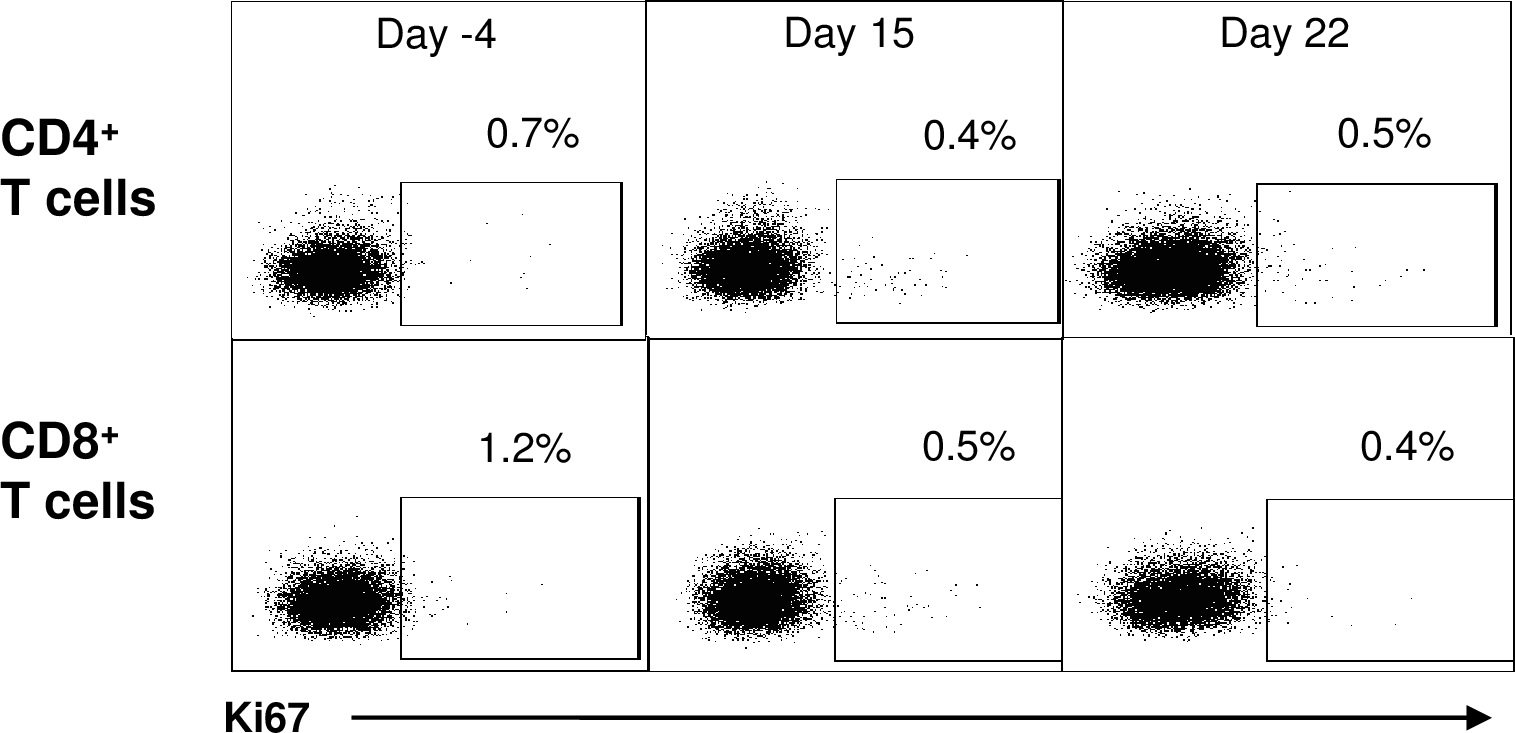

Supplement: S8 Fig — Representative dot plots of CD4+ and CD8+ T cells isolated from PBMC from cynomolgus macaques treated intravenously with 10 mg/kg of AGEN1884. PBMC were analyzed for Ki67 expression in CD4+ and CD8+ T cells at four days prior to treatment or 15 and 22 days after treatment. (TIF) [file pone.0191926.s009.tif]

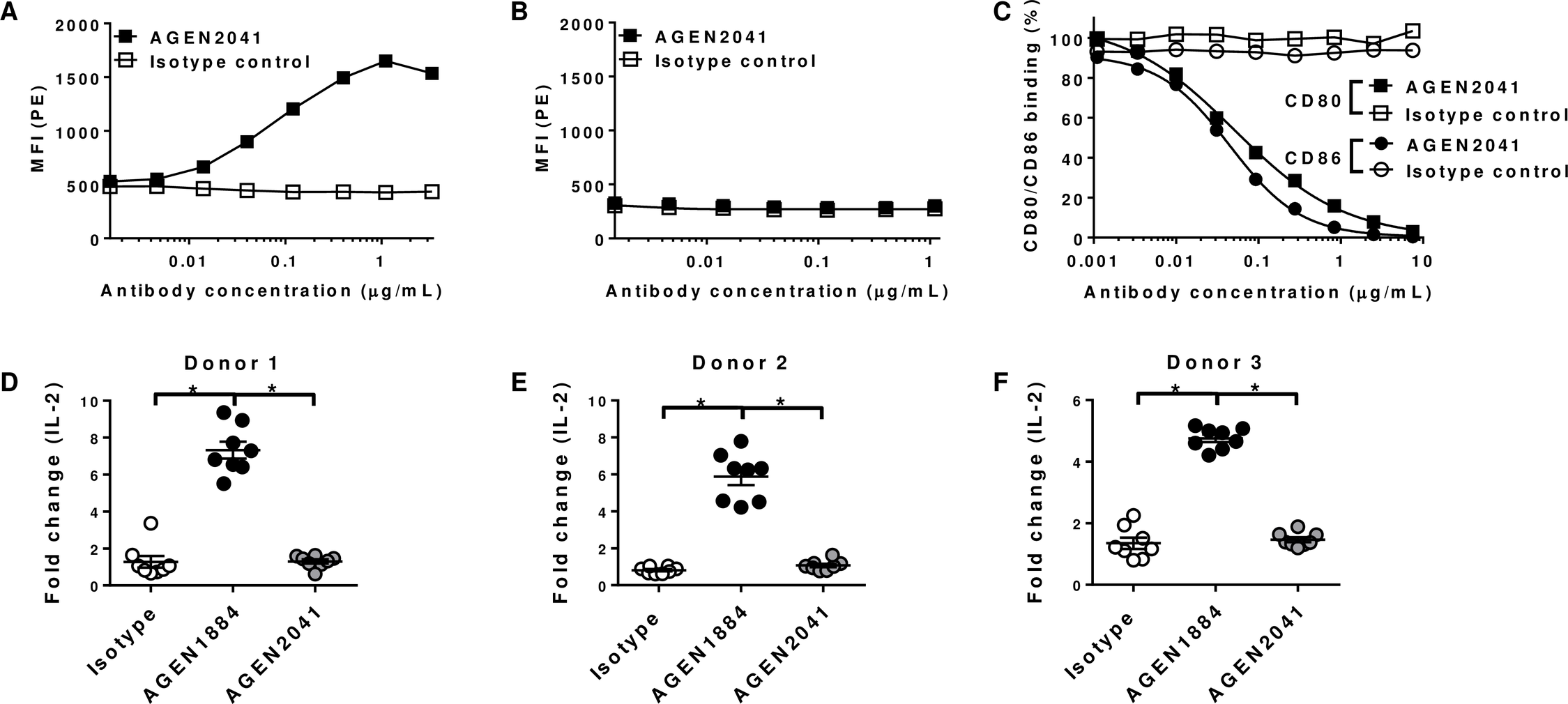

Supplement: S9 Fig — (A-B) AGEN2041 binding to a (A) Jurkat cell line genetically engineered to express human CTLA-4 or (B) wildtype (CTLA-4-negative) Jurkat cell line. (C) Binding of fluorescently-labeled CD80-Fc or CD86-Fc (1 nM) in the presence of increasing concentrations of AGEN2041 or an IgG2 isotype control. Binding to CTLA-4-linked microspheres was assessed using Luminex. (D-F) Primary human PBMC were stimulated with a sub-maximal concentration of the SEA peptide (100 ng/mL) and a single dose of AGEN1884, AGEN2041 or an isotype control (10 μg/mL). Representative data indicate the mean ± SEM of multiple replicates in each treatment group (n = 3). Data were analyzed using a Student’s t-test. Significant differences depicted were p<0.01 (*). (TIF) [file pone.0191926.s010.tif]

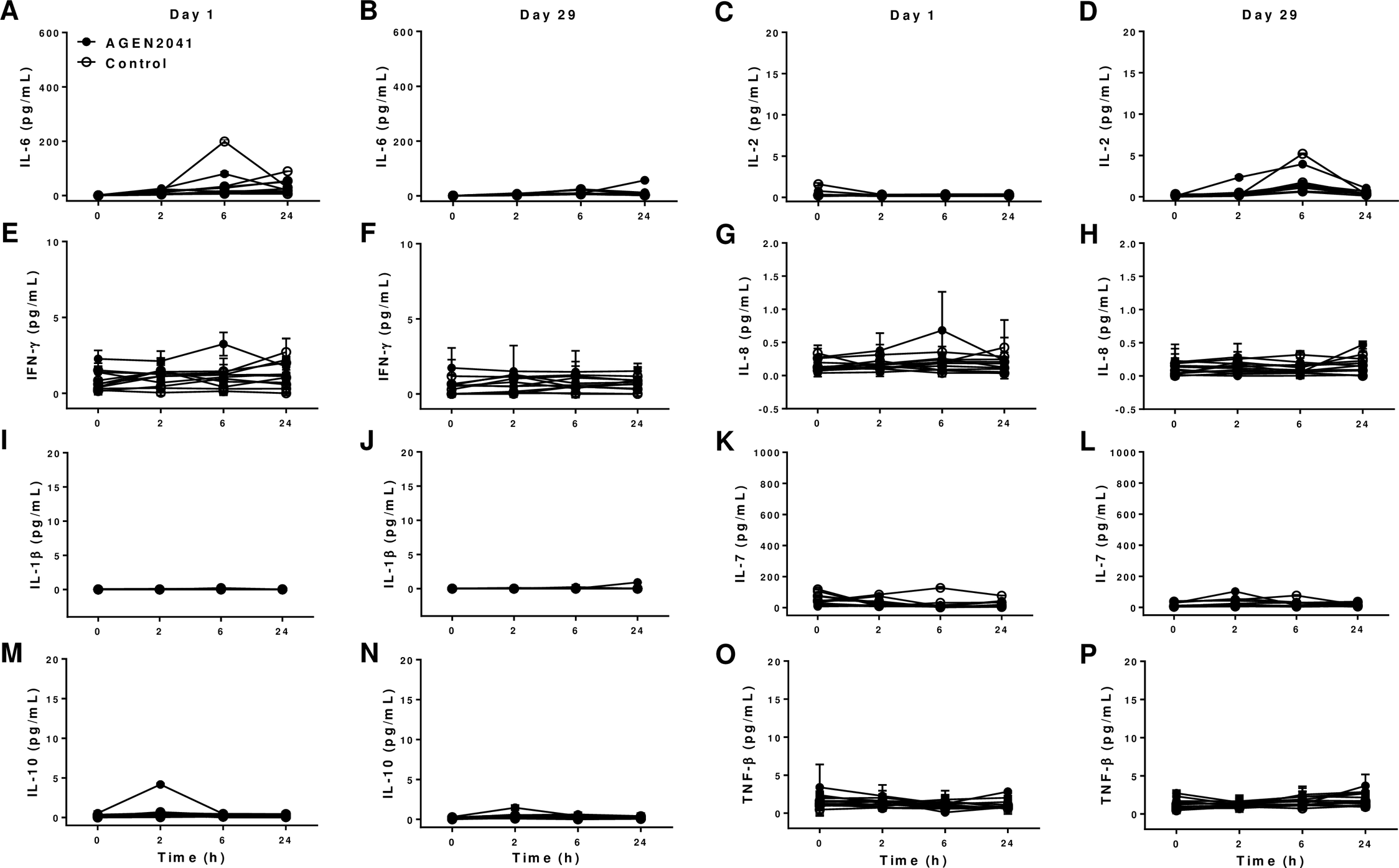

Supplement: S10 Fig — Serum cytokines (A,B) IL-6, (C,D) IL-2, (E,F) IFN-γ and (G,H) IL-8, (I,J) IL-1β, (K,L) IL-7, (M,N) IL-10 and (O,P) TNF-β were measured pre-dose (0 hrs) and 2, 6 and 24 hrs post-infusion at (A,C,E,G,I,K,M,O) day 1 and (B,D,F,H,J,L,N,P) day 29 from two groups of cynomolgus macaques (n = 6 per group) vaccinated with 10 mg/kg of AGEN2041 or a control vehicle in addition to a KLH and HBsAg vaccine. (TIF) [file pone.0191926.s011.tif]

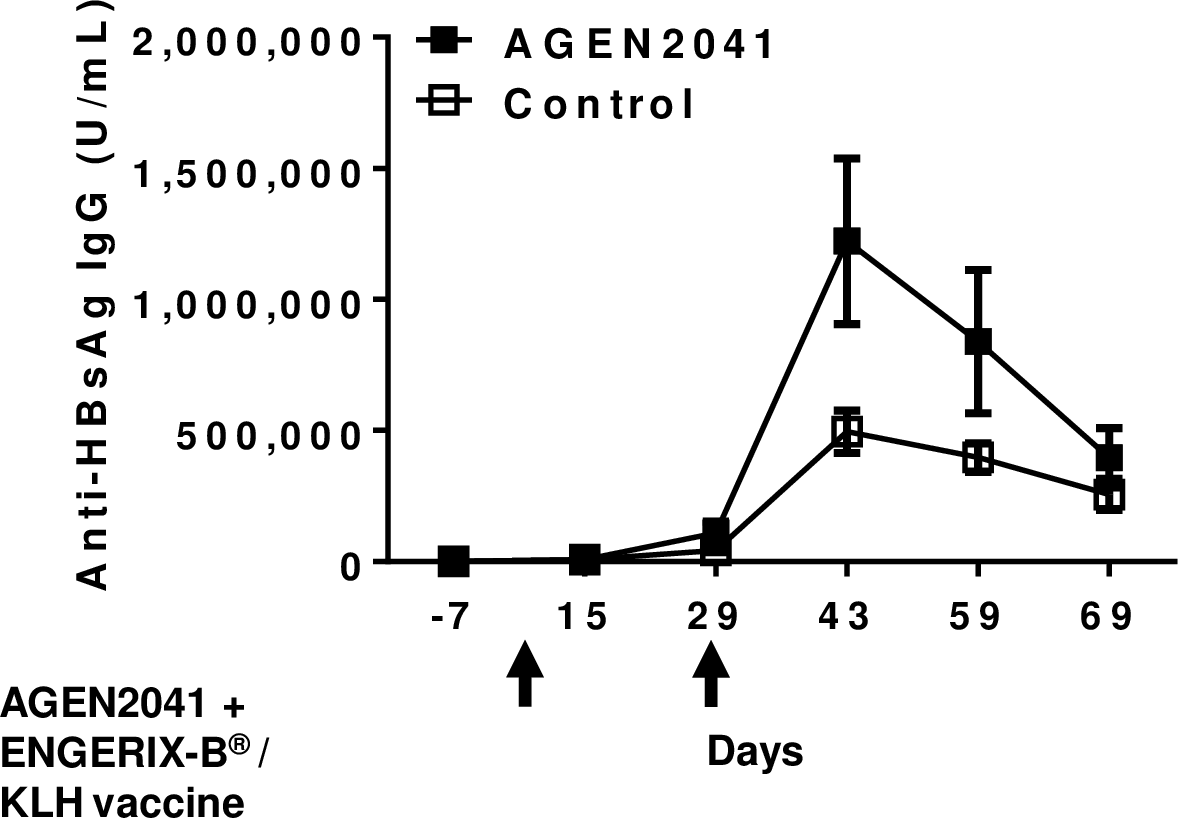

Supplement: S11 Fig — Cynomolgus macaques (n = 6 per group) were administered 10 mg/kg of AGEN2041 via intravenous (IV) administration with an ENGERIX-B® and KLH vaccine on days 1 and 29. Duplicate samples were analyzed for anti-HBsAg-specific IgG serum titers in PBMC. (TIF) [file pone.0191926.s012.tif]
